# Spirit of the Foreign Periodicals

**Published:** 1835-04-01

**Authors:** 


### Spirit of the Foreign Periodicals, &c.

### State of Medical Opinion in Paris.

In the following brief remarks, we shall attempt to embody the general scope and tenor of the introductory lectures, recently delivered by three of the most eminent professors, MM. Velpeau, Chomel, and Andral, on "Clinique Chirurgicale, Clinique Médicale, et Pathologie Interne," before the Faculty of Medicine in Paris. "Three doctrines," says the first of these authors, "have successively swayed the opinions of medical men, viz. those of vitalism, humorism, and solidism. In the present day, vitalism, as a system of tenets, is almost banished from the French school of physiological medicine; but still retains its ground in Germany, and has also a sanctuary in the faculty at Montpelier. Humorism, too, like its predecessor, had, until within the last 12 years, experienced with us the same fate, and every system was thoroughly imbued with the spirit of an exclusive solidism. Nevertheless, the one has always appeared to me to be quite as rational, and as harmonizing with the phenomena of living matter, as the other; and it is now many years since I first raised my voice in support of a limited humorism, although, at that time, it required a certain degree of courage to call in question the authority of the reigning doctrines. My opinions have, however, been gradually advancing in favour, and their truth is now acknowledged to be confirmed alike by reasoning and by observation. Does not the body consist of fluids as well as of solids? are not all the solids originally, and indeed unceasingly, derivable from the fluids? and can it, therefore, be at all wonderful, that the latter may be primarily diseased as well as the former? Certainly not. Surely, then, it must be illogical in the extreme, to refuse to recognize any but secondary or consecutive diseases of the fluids. The well-known results of transfusion and inoculation abundantly testify the operation of direct changes of the circulating fluids, and one very interesting feature of such experiments is, that in numerous cases, the same disease is generated in the person on whom they are performed, as affects him from whom the translated matter is obtained. Besides this important set of humoral, or at least of partially humoral, diseases, there is another class, which, until of late years, has been totally overlooked since the beginning of the present century—we allude to the spontaneous alterations of the blood, independent of the introduction of any foreign matter from without, and to those alterations particularly, in which there is found a purulent admixture in the bloodvessels. It was in 1818 that my attention was first drawn to this curious morbid phenomenon:—A woman in the hospital at Tours died, from the effects of a comminuted fracture of the tibia, complicated with numerous depositions of pus in different parts. On dissection, the principal viscera were found 'parsémés' with small abscesses, the walls of which exhibited no traces of any inflammatory action having ever existed; and the blood exhibited a very altered appearance, as if purulent matter had been mixed with it. "Ce fut un trait de lumière."—(*Vide* a remarkable case of this morbid change in the subsequent article of the present No.) Since that time, the attention of many medical men has been drawn to this subject, and numerous cases have been published in the various journals. The researches of MM. Dance, Lee, &c. have shewn that, in some fatal puerperal diseases, the contents of the uterine and other veins not unfrequently exhibit very striking abnormal appearances, purulent matter being blended with the altered blood; and in cancer, melanosis, &c. portions of encephaloid substance have been found in the veins after death. We do not, however, lay much stress on the pathological changes of the blood, in the set of cases now alluded to, as they are indubitably consecutive and secondary, not primary and idiopathic, in their occurrence.

But it is unnecessary to argue, at greater length, in favour of a limited, not an exclusive, humorism. The true physiologist omits the consideration of no part of the living body, and of no agent which can influence its operations and power; and, well knowing the complexity of the machine, he is forced to admit that diseases "sont des problèmes infiniment complexes, dans la solution desquels il faut faire entrer tous les elemens de l'organisme." Every morbid phenomenon implies the existence of a heterogeneous agent, which, either derived from without or generated within the body, is the exciting cause of the diseased process. The effects of this agent vary, according to its locality, quantity, and especially its nature. We may mention, as undisputed examples of the obvious operation of this agent, the consequences of exposure to miasms and certain viruses. Now this etiological doctrine, admitted to be true in the case of some diseases, and rendered so probable in that of others, leads the philosophic physician to enquire whether there are any therapeutic specifics. It is in vain to expect that an incipient small-pox, ague, or itch, can be cut short by any antiphlogistic remedies—the "peccant thing" will resist all such attempts until it either exhausts itself, after continuing for a certain time, or it is counteracted by a specific remedy. Let it not be imagined that we deny the occurrence of an inflammatory process, in these and other cognate diseases; all that we contend for is, that this process is not an original and essential, but only a secondary and superinduced attribute of them. The abstraction of blood may be of the greatest benefit in checking or allaying increased or irregular action, but it does not and cannot have any direct influence in combating the primary cause of the disease; "elle ne s'addresse qu'aux epiphenomènes." In not a few cases, the body is of itself capable of resisting and overcoming the operation of the noxious agent, and then it is either nullified, or expelled from the system by its own unassisted reaction; but these cases are rare, compared with the multitude of those in which the natural powers are insufficient, and against which we have not yet discovered any directly neutralizing remedy. We are, therefore, in default of this knowledge, obliged to be satisfied with employing the "rational method" of prevention and cure, in our treatment of diseases, by combating symptoms as they are developed, or even by merely employing means which have been found, by experience, to be remedial, although we cannot explain the why and the wherefore of their operation.

The pervading tone of Professor Chomel's lectures exhibits an equally enlarged and philosophical view of medical science as that now unfolded by M. Velpeau. Unfettered by the dogmata of any one school, and with a liberal and comprehensive turn of mind, which leads him to view things and appearances as he finds them, and not as he wishes to find them, he hesitates not to expose the fallacy of the prevailing exclusivism, which has tyrannized in medicine during the last thirty years. "Morbid anatomy may be useful; nay, it is (says he) indispensable in the study of diseases: it is one of the most solid bases of rational diagnosis, and one of the surest guides to a rational system of therapeutics; but it is not, for all this, the only and the exclusive one. Its importance has been viciously exaggerated of late years, and the organic pathology has, perhaps, led to quite as much mischief, in doctrine and practice, as the undefinable vagaries of the old school of vitalism which preceded it—"le premier ne prouve souvent rien quant au traitement applicable; car l'etat general de l'individu, l'ancienneté, l'intensité, la physionomie des symptomes, entrent souvent, comme elemens essentiels et primordiaux, dans l'indication, et, pour ainsi dire, dans la construction de la maladie tout entière." The lesions revealed by dissection point out the effects, but surely not the causes of disease, and, in too many cases, contribute but little to the discovery of a successful treatment. To observe scrupulously—to be cautious of making deductions, except from facts minutely and frequently recorded—to avoid framing hypotheses, and indulging in quick conjectures, these are the elements of true medical philosophy. Every mere system of physic must necessarily be imperfect and full of faults; and, although the simple study of appearances, by themselves, has the effect of rendering a pursuit teasingly complicated and laborious, it is the only safe course to follow in so doubtful a science as medicine ever must be. When we begin to reason as to the causes, and the essence or nature of any particular disease, we enter on a labyrinth of multitudinous perplexities. To say that such an organ or viscus is affected, is vaguely to hint at the locality of the mischief; to indicate the particular tissue involved, is to circumscribe the allegation within narrower bounds; to describe the state of the sanguiferous and other vessels is, we must confess, to advance a step further to the object of our search; but still, beyond this rude knowledge there are other morbid elements, whose nature and whose workings are almost wholly hid from our ken. It is by no means improbable that some of the imponderable gases, electricity and galvanism, for example, play an important part in the generation of morbid processes; and that chemistry may, in the course of time, be made a more successful analyst, than hitherto, of pathological changes, by revealing the alterations in the immediate principles of the organized body, at the beginning and during the progress of a deviation from health. Have we not some well-grounded reasons for believing, that an excess or deficiency of one or other of the elementary constituents of the blood may be connected with the development of some diseases? that, for example, an excess of nitrogen may predispose to, or virtually induce, a deposition of uric acid in the kidneys and in the joints, and thus give rise to gravel and gout? Is it improbable that the accumulation of fatty matter, in the livers of phthisical patients, may be dependent upon the deficient elimination of an hydrogenised principle from the lungs? and may we not suspect that chlorosis coincides with a diminution of the normal quantity of iron contained in the blood? Speculations of this nature may seem visionary to those who call themselves practical men; but they may nevertheless be founded in truth, and may, at a future period, affect the practice of our profession in an essential degree. The time may, indeed, be distant, when medical chemistry may achieve results so important; but we may hope to prepare the way, at least, for such enquiries, by a more frequent and minute examination of the more immediate and obvious constituents of the blood, viz. the albumen, fibrine, &c. in health and disease, than has yet been done.—"Quoiqu'il en soit, les solides et le sang, voila les deux bases, les deux supports d'une maladie, elemens inseparables, alternativement cause et effet de leurs mutuelles alterations." The one cannot be long affected with disease, without the other being speedily involved."

Such are the sentiments of M. Chomel; and we come now to consider those of a still higher authority than even him, or, indeed, than almost any other of the French school. The pathologist who takes so enlarged views of medical science as M. Andral does, could not long fail to perceive the errors of the reigning French school, which endeavoured to localise every malady, and strove so earnestly to banish such epithets as general, systemic, or essential, applied to diseases. Although led along, for some years, with the current of public opinion, he speedily discovered how ill it harmonized with clinical observations, and he was forced to recognize the existence of diseases essentially and primarily general, or in which the whole organism is disturbed from the first moment of invasion, as well as those which are primarily local, although the system may soon be forced to sympathise.

The ancients committed the error of regarding almost all diseases as general; the moderns have gone to the other extreme, and have been equally blind to Nature's operations, by refusing to admit any as such. Not to mention the numerous class of fevers, what shall we say of scrofula? in what exclusive part of the system can it be said to be localised? It is, in truth, essentially a constitutional disease. As it may be interesting to the English reader to know the classification of diseases which M. Andral adopts in his lectures, we have subjoined the following table.

| | | |
|---|---|---|
| 1. Lesions of the Circulation | Hyperemia .......... | Active; Passive; Mechanical |
| | Anemia | |
| | Phlegmasia | |
| | Hæmorrhage ........ | Active; Passive; Mechanical |
| 2. Lesions of Secretion.... | Of the liquids.... ... | Excess—dropsy, flux; Diminution; Alteration |
| | Of the gases ........ | Alteration in quantity; Alteration in quality |
| 3. Lesions of Nutrition.... | Errors of conformation | |
| | Alterations in texture.. | Hypertrophe; Atrophy; Perversion |
| | Cessation (gangrene) | |
| | Accidental productions | Abnormal structure; Entozoa |
| 4. Lesions of Innervation.. | Lesions of sensibility | |
| | ——— of motility | |
| | ——— of a special function, as gastralgia | |
| 5. Lesions of Function .... | These necessarily imply a material alteration in some organ or another; but an alteration whose essence is unknown, and which is distinct from the lesions of the four preceding classes. Epilepsy, chorea, dyspepsia, &c. may be adduced as examples. | |

---

### PURULENT ALTERATION OF THE BLOOD.

A woman, 27 years of age, who was recently admitted into the Hôpital de la Pitié, under the care of Professor Rostan, presented the following symptoms:—The general emaciation was extreme—the breathing hurried and difficult—the pulse rapid and very weak—the bowels were much purged, and she had profuse perspirations, especially when asleep. From the imperfect account which could be obtained from herself, it appeared that she had been long ill, and had, within the preceding fortnight, been confined to bed. In such a case, it was natural to suspect the existence of confirmed phthisis, and an attempt was, therefore, made to examine the chest by auscultation; but this was unsatisfactory, as the patient could not remain quiet for a moment, and she was continually moaning. She died on the second day after her admission.

*Dissection.* On examining the encephalon, all the sinuses were found to be filled with a thin, reddish fluid, in which numerous grey-coloured and friable flocculi floated. The contents of the veins of the Pia mater at some points were similar; at others, they had a more decided purulent appearance. The parietes of these vessels did not exhibit any change of texture. The lungs were on the whole healthy; no vomicæ, nor even tubercles could be discovered in any part; the bases of the lobes were gorged with blood, but not hepatised. The bronchi contained a quantity of mucosity, but their surface was not reddened, or otherwise abnormal. The heart was large, and excessively distended; and more especially its right cavities. On cutting into the ventricles, a quantity of blood, having the colour of wine-lees, escaped; on examining this blood, it was observed that it contained several dark-coloured softish coagula, and also an immense number of yellow friable flocculi, exhibiting all the appearances of half-concreted pus, and resembling the semi-purulent flocculi found in the abdomen after peritonitis. In the left auricle, one of these flocculent masses, as large as a walnut, was found floating free in the decomposed blood. The contents of the pulmonary artery and veins were altogether similar. No trace of disease could be discovered on the inner investing membrane of the heart and large blood-vessels, except that it was perhaps unusually pale. On opening the thoracic and abdominal aorta, the carotid artery and jugular veins, similarly altered blood, with purulent flocculi floating in it, was found in their cavities.

*Abdomen.* The stomach and small bowels did not present any marked appearances of disease; the glandulæ Peyeri were healthy. Almost the whole extent of the large intestines was thickened, and their mucous coat exhibited numerous ulcerated points; these minute ulcers were superficial, and could be best seen by stretching a piece of the gut, and holding it up between the eye and the light: in the cæcum they were largest and also deepest. The mesenteric glands were very generally swollen, some of them being as large as walnuts; when cut across, their structure was not unlike that of hepatised lung, but no traces of suppuration could be detected. The mesenteric arteries and veins contained a mixture of blood and purulent matter, such as we have described, as observed in other parts of the sanguiferous system: the parietes of these vessels appeared nevertheless to be quite sound. The liver, and still more remarkably the spleen, were of enormous size, and their texture was much indurated. The latter viscus exhibited numerous white nacreous points, and its colour throughout was rather a deep yellow (not unlike to that of some livers) than the usual dark red. The hepatic and splenic blood-vessels were filled with "le liquide couleur lie-de-vin, et ces flocons purulens deja mentionnés." The kidneys, bladder, uterus, and ovaries were in a normal state.

*Blood-vessels of the Extremities.*—"Partout nous avons rencontré la meme alteration du liquide qu'elles contenaient. Nous avons ouvert plusieurs vaisseaux collateraux des doigts, et l'alteration du sang existait là comme partout ailleurs. Dans aucun point nous n'avons trouvé de traces d'inflammation, soit dans les arteres, soit dans les veines: tous ces vaisseaux ont été incisés, leur face interne a été lavée et examinée avec le plus grand soin, et partout elle etoit aussi intacte que dans les autres organes de la circulation."

In concluding the report of this most interesting dissection, we cannot help expressing our regret, that the large joints were not examined, as it is well known, that in some cases, similar to the one now described, very extensive depositions of purulent matter have been found in these cavities. The omission arose altogether in the present instance from forgetfulness. It deserves however to be noticed, that the patient, although in a dying state when admitted into this hospital, made no complaint of pain or uneasiness in any of her joints.

*Remarks.* Perhaps in no case, hitherto recorded, has there been found so general and so marked an alteration of the circulating fluid as in the one which we have now described. In some of them there was found purulent matter mixed with coagula in the venæ cavæ, and in the right cavities of the heart; in others, but these are more rare, the admixture was discovered in the ramifications of the pulmonary artery; and when it was observed in the aorta, it was always in very minute quantities; a few drops only of pus being detected in the centre of largish coagula of blood. The extremely interesting feature of the present case is the universality of the morbid change; and that, notwithstanding this, there was no appreciable lesion of the blood-vessels in any part of the body. The examination of this point was most minutely made: all the vessels of the extremities, the sinuses of the dura mater, the arteries of the brain, all the considerable vessels of the chest and abdomen were not only opened, but carefully washed, so as to free their surfaces "de la matiere sanieuse qu'ils renfermaient;" but no vestige of morbid alteration was to be seen in any of them; not even a patch of inflammatory redness. This entire absence of such changes is the more worthy of remark, as in almost all the cases hitherto recorded, there has been discovered after death, at some part or other of the venous system, the mark of a pre-existent phlegmasia, or of its consequences, such as pseudo-membranous effusion, thickening and hardening of the vascular parietes, or injection of the vasa vasorum, &c.; yet in none has there been found even a moiety of the purulent matter in the blood. The conclusion is therefore forced upon us, that the proximate cause of the morbid change was not an inflammation of the vessels, either arteries or veins, although we certainly did, during the life of the patient, suspect the existence of uterine phlebitis, from having seen some cases of this disease, in which the symptoms were analogous to those in the present case: the dissection, however, completely disproved this idea. It may be supposed by some that the large quantity of purulent matter which was found mixed with the blood had been absorbed from some internal abscess, and been thus conveyed into the circulating system; and although no abscess was discovered in any part examined, it may possibly be conjectured that the articulations were the seat of the mischief, and that it was into their cavities that the purulent effusion had originally taken place. True it is, that we cannot positively contradict this opinion, from the joints not having been opened; but the supposition appears to us very problematical, and we should be inclined to suppose, that the pus, if it really did exist in the present case in the joints, was the result, rather than the cause, of the alteration of the whole mass of the blood. It is now an acknowledged fact of pathology, that when a vein becomes inflamed, or is exposed with open mouth to a purulent deposit, the joints, even the most distant from the seat of the disease, are often found to contain pus in considerable quantities. In uterine phlebitis, and after amputations and other important operations, this phenomenon has been frequently observed. In some of these cases, the existence of suppuration, accompanied perhaps with softening and even ulceration of the articular cartilages, was associated during life with symptoms so obscure and unsatisfactory, that the most experienced practitioners, even when their suspicions were excited, were left in doubt and uncertainty; whereas, in other cases, the symptoms were sufficiently well-marked, such as excruciating pain in the parts, outward redness and swelling, &c.

We have already stated that our patient made no complaint of uneasiness in any of the joints. In conclusion, the most interesting features of the present case may be stated to be the universality of the purulent admixture with the blood, the absence of any visible disease of the blood-vessels, and the absence of internal abscesses: and we shall now leave it to our readers to determine whether the disease was attributable to a primary and idiopathic disease of the circulating fluid, or whether the morbid change which this had suffered was secondary and consecutive. "Peut-etre," says M. Andral in the fourth vol. of his Clinique Medicale, "l'epoque n'est elle pas eloignée où l'on reviendra à cette idée de De Haen, qui admettait que, dans certaines circonstances, le pus peut se former de toutes pieces dans le sang comme on voit s'y former, l'urée dans l'état physiologique." Those who are interested in following out the curious subject of purulent changes of the blood, will find much instructive matter in Velpeau's memoirs, published in the Archives Generales and the Revue Medicale for 1826; in M. Dance's paper in the Archives for Dec. 1828, and in some observations communicated by M. Le Gallois to the Journal Hebdomadaire for May, 1829.—*Archives Gen. Oct.* 1834.

---

### Cases of Traumatic Phlebitis.

*Case* 1. A man, 25 years of age, was brought to the Hotel Dieu, in consequence of a lacerated wound of the forehead, which he said he had sustained by falling out of a cabriolet. The lacerated and contused appearance however of the wound, made M. Dupuytren suspect another cause for the injury; and upon a rigid examination, the patient at length confessed that he had, during a moment of mental disquietude, pointed a pistol to his head, and that the two balls, with which it was loaded, had torn and lacerated the integuments. The frontal bone could be felt denuded and rough on the surface. The wound was enlarged, to remove the existing tension of the parts; and a free venæsection and other antiphlogistic means were then actively employed. Erysipelas however attacked the wound, and spread over the face and scalp; symptoms also of gastro-enteritis came on. They were treated by leeches and the internal use of the tartrate of antimony. The suppuration from the wound became very profuse, and the unfavorable symptoms of universal depression, delirium, subsultus tendinum, &c. indicated a fatal result. Coma supervened, and he died at the end of the third week after his admission into the hospital. (It appears from the report that on the two days preceding his death, and when the train of last-mentioned symptoms had fairly set in, the use of large numbers of leeches and of full doses of the antimonial tartrate had been continued.—Surely *such treatment* was most unwise and pernicious.)—*Rev.*

*Dissection.* The scalp was found to be separated from the bone all round the edges of the wound to the extent of an inch or two; at one part the periosteum was found detached, and the surface of the bone quite bare. The adjacent soft parts "contenaient du pus non rassemblé en foyer." Under the eyebrow several drops of pus were observed to ooze out, as if they escaped from the divided blood-vessels. The veins of the orbits were found to be full of pus. When the scull-cap was removed, a deposit of purulent matter was found between the bone and the dura mater, and also underneath the arachnoid coat, opposite the seat of the injury. The substance of the cerebrum, at this part, was softened and discoloured, and the neighbourhood was redder than usual, and its vessels fuller of blood. The liver was "parsemé" with yellow patches, and the surface of each of these patches was covered with a deposition of coagulable lymph. On dividing it across, numerous abscesses were observed throughout its structure, varying in size from that of a pea to that of a walnut. The number of these "foyers purulens" must have been at least 150. Both lungs exhibited similar morbid changes; but in them the abscesses were more diffused, probably in consequence of the pulmonary interstitial cellular tissue being looser and less compact than that of the liver.

*Case* 2. A man, 37 years of age, was admitted into the hospital, with a comminuted fracture of both bones of the left leg; this injury was complicated with five or six narrow wounds of the soft parts, and had been caused by a heavy weight having fallen on the limb. The patient was bled no fewer than six times from the 28th of January (the date of the accident) to the 7th of February. "Son etat n'offrit rien de particulier pendant ce temps." (Where then the necessity of such vigorous treatment?) But on this day he complained more than he had yet done of severe head-ache, and towards the evening he had a feverish attack, commencing with a shivering fit, and accompanied with tendency to delirium. On examining the bandages round the leg, they were found to be moistened with an offensive discharge; and when the limb was exposed "l'appareil fut trouvè baigné de pus et de sanie, les compresses trempées d'un pus noiratre, la peau tombée en suppuration, en gangrene là ou elle etait le plus contusé."

Considering such a state of things, (arising, it would seem, from sheer neglect,) we cannot be surprised to be told, that "des lors, l'etat general du malade presenta plus de danger que l'etat local." The pulse became frequent and feeble; there was a tendency to profuse perspiration; a troublesome cough and frequent expectoration came on; the breathing was oppressed, and the features indicated great inward distress. At one time, the patient was muttering and incoherent in his speech, and at another, he was almost quite comatose. A camphorated blister was applied to the chest; but neither this, nor the julep of polygala, nor indeed any other julep, was or could be of the least use. The pòor fellow died on the 12th of the month.

*Dissection.* The seat of the fracture was found bathed with pus; the veins of the limb were most carefully examined, but no traces of purulent matter could be discovered, either in these, or in any of the other veins of the body. On opening the thorax, the surface of the lungs was "parsemé" with at least a score of yellow patches, the average size of which was about that of a horse-bean. When the substance of the lungs was divided, these "taches jaunes" were found to be composed "de petits grains extremement fins, jaunes, d'une consistence assez forte, se rapprochant de celles de la graisse." On squeezing them, globules of pus oozed out. The liver exhibited a similar, only a more extensive degeneration. At five or six places on the surface of the right lobe, there were abscesses, some of the size of the walnut, and others as large as a hen's egg; the investing peritoneal coat was thickened and opaque. "Le rest du corps presentait un etat d'anemie tres marqué." (No wonder, when we think of the patient being bled "six fois" within ten days after the accident.)—*De la Phlebite Traumatique. Thes. Paris,* 1833.

---

### On the Nature and Treatment of Acute Rheumatism.

There are at present two prevalent theories in reference to the etiology of this very common disease, and it is not a very easy matter to decide on their respective merits, or whether either of them is exclusively and 'per se' correct: According to the opinions of one set of physicians, acute rheumatismal arthritis depends upon a primary alteration of the blood, its fibrinous portion being considered to be either superabundant, or at all events less intimately blended, and therefore more readily separable from its other constituents; whereas the opposite party deny that the change in the blood is invariable and uniform, and regard rheumatism as simple inflammation of the articular fibro-serous textures, which may be limited to one joint, or may effect several at a time, in consequence of the very intimate sympathy which exists between every part of the sero-fibrous system. It is not our intention to enter upon the discussion of the respective merits of these two doctrines, nor yet to analyse the leading phenomena of the disease: At present we shall confine our remarks to the consideration of an occasional symptom, which of late years has drawn the attention of many medical authors, we mean the irritative affection of the heart. When a rheumatic patient is seized with this affection, he experiences deep in the cardiac region sharp shooting pains, and at other times a dull and constant uneasiness, varying indeed in different cases, as to severity and as to the exact seat of the distress.

These feelings are usually accompanied with an irregular tumultuous action of the heart; its pulsations are so much more rapid and more forcible than in health, that if the ear be applied for the purpose of auscultation, the physician may be much inclined to suppose that the organ has already become hypertrophied. In by far the greater number of cases, the symptoms now enumerated cease after a few days continuance, and then it will be found that the heart when examined with the ear, has resumed its natural tranquil state.

What is the nature or cause of these cardiac pains? Most medical men will answer that they are essentially rheumatic and depend upon a rheumatic affection of the substance of the heart itself. The advocates of the humoral etiology of the disease explain the occurrence of the cardiac pains, 'en disant que, puisque cet organe (le cœur) est penétré d'un sang altéré, il n'y a rien de surprenant qu'il eprouve une affection semblable à celle qui sevit sur les articulations,' whereas the solidists attribute them to a sympathy of the heart with the other parts of the muscular and serous systems, and to a consequent metastasis of the morbid act from one part to another. It is to be observed, that although we have classed together the muscular and serous systems, we believe that rheumatic affections of the heart are very generally seated in the membranous, and not in the fleshy tissue of the heart; in short that pericarditis is infinitely more frequent than genuine carditis. Reasoning a priori might have led to the same conclusion, on the ground, that in acute rheumatism the fibrous tissues of the joints are much more commonly affected than the surrounding muscles, and that therefore the membranous investitures of the heart would more probably be involved in a translation of the disease, than the muscular fibres.* The rapid disappearance of the disease, and the very speedy cessation of all the cardiac symptoms, coupled with the occasionally obscure character of these, while they last, lead us to the same conclusions.

In reference to the treatment of acute rheumatism, Dr. Barthelemy (author of this communication), who is an assistant in Broussais' Hospital, the Val de Grace, seems to suppose that the only genuine and heroic remedy against acute rheumatism is the detraction of blood; and discarding therefore the consideration of the other curative means, he limits his remarks to the enquiry, whether general, or local bloodletting is most beneficial in this disease. The modern humoral (using this epithet in the meaning explained at the beginning of this paper) pathologists prefer the former practice, while the 'medecins physiologistes' have much more faith in repeated leechings. M. M. Roche and Bouillaud are in the habit of bleeding their patients, three, four and five times successively, and at each time to the amount of 16 or 20 ounces: one 'pauvre malade,' who had the misfortune to be cured of acute rheumatism by eight copious venæsections, seems to have excited in an especial degree Dr. B.'s sympathy: he went, he tells us, to see him from mere curiosity (a curiosity which reminds us of that of the Scotch judge, who in fun condemned a prisoner, 'that he might see how the b——r would look!'); 'selon toute apparence, il a dû rester foible et decoloré pendant plusieurs mois, &c.' Dr. B. although hostile to the heroic use of the lancet, approves of one or two general bleedings in most cases of acute rheumatism, and follows up this antiphlogistic treatment by repeated leechings. This is the treatment which has been adopted with such signal success by MM. Piorry and Broussais.

On perusing a subsequent number of

* It is altogether a most gratuitous assumption, and an assumption too, which is contradicted by accurate enquiries, to assert that 'rheumatisme articulaire aigu' (the rheumatic fever of some authors) is seated in the muscular tissues: it is to the synovial membranes, what pleurisy is to the pleura, peritonitis to the peritoneum, &c. and indeed these latter diseases have sometimes been designated pleuritic and peritonitic fevers.—*Bouillaud.*

the same hebdomadal, we find a letter from M. Roche, in refutation of M. Barthelemy's account of the 'pauvre malade' for whom he had shewn so much sympathy: 'par miracle sans doute,' says M. R. 'il n'est resté, ni foible, ni decoloré pendant plusieurs mois; il n' a pas été bouleversé pendant plusieurs années; il n'est pas devenu hydropique; il a pu digerer des le premier jour de sa convalescence; enfin une vingtaine de jours apres sa guerison il avait repris son metier de batteur d'or.'

Among the novelties of medical literature, may be mentioned the extraordinary notion of Professor Hildenbrand of Pavia, that rheumatism is 'le resultat d'un defaut d'equilibre survenu entre la chaleur et l'electricité du corps, et celle de l'atmosphere.' Acting on this principle, he orders all the affected parts to be well covered with 'corps idio-electriques, tels que le colon, la flanelle, le taffetas gommé, prealablement imbibés de substances resineuses.' As a matter of course, his success has been truly astonishing!!

Professor Bouillaud, also, in his recent clinical lectures, has alluded to M. Barthelemy's paper, which he says, contains some assertions respecting his practice, which are inexact, and require therefore to be answered. The Professor is of opinion that M. B. has failed to adduce any proof, that general bleedings ought seldom to be practised more than once or twice in cases of acute rheumatism, or that they can be superseded with advantage by leeching or cupping; and he alludes to the results of his own clinique at the La Charité, to shew that venesection, even to the sixth or seventh time, is by far the speediest method of curing the disease, and that such a vigorous practice is by no means usually followed 'par une tres longue convalescence, faiblesse, anemie, infiltration, ni par aucune de ces graves terminaisons dont nous menace notre confrere.'

The following resume of a few cases will give some idea of Bouillaud's practice.

A patient was labouring under severe rheumatism of one knee, when he applied for relief. He was twice cupped, each time to the amount of 12 ounces, and had 30 leeches applied over the joint; all in the course of three days: compression and mercurial inunction on the part was then employed, and the treatment 'fut terminé' by another ample leeching: the cure was complete in 17 days.

In the second case, the man had suffered for eight days from rheumatism of the right foot, left knee and right shoulder: He was bled, 'coup sur coup' three times, and cupped once (3 palettes, or about 12 oz. at each time); then compression and mercurial frictions were used:—cure in six days.

The third case was that of a young man, in whom the affection was fixed in the two wrists; it was 'd'une extreme tenacité;' for in spite of being bled from the arm seven times, and being also leeched and cupped, not to mention the use of baths, narcotics, internally and externally, mercurial frictions and compression, 'il n'etait pas completement gueri lorsqu'il demanda sa sortie, apres trois semaines de sejour à l'hopital.' The Professor very adroitly adds 'que le rheumatisme aigu fixé aux poignets, est plus rebelle au traitement que partout ailleurs.'

A man had experienced an attack of general rheumatism for some time before his admission into the hospital; the complaint remained fixed in the right wrist and left knee. On the first day he was bled to twenty oz. and on the following two days, to 12 oz. each time. On the fifth day, after his admission, 'il etait deliveré de toutes ses douleurs, qui n'ont pas reparu.' The fifth patient was bled twice, and had 20 leeches applied: cure in eight days.

The sixth patient was bled twice, and also cupped twice: cure in 12 days.

The seventh, a healthy and plethoric woman, affected with general acute rheumatism, was bled 7 times, (8 oz. each time) in thirteen days, and was cured by the 16th day.

Such are the data, (selected from an immense number of analagous cases) by which Professor Bouillaud attempts to prove the speedy and decided efficacy of vigorous depletions of blood against acute rheumatism.—*Journal Hebdomadaire.*

### On the Insect of the Itch—(Acarus Scabiei.)

The "chasse" of this most despicable vermin was, some two or three months ago, an all-absorbing occupation of the medical savans in that most enlightened city of the world, Paris—la belle Paris. The ardour of the huntsmen was quite commensurate with the importance of the game; many a microscope was bought, many an eye was strained in this great emprise, and many a pen was wielded, to tell all the wonders seen or imagined. Every journal teemed with descriptions and counter-descriptions, with notes of discoveries, letters of reply, reclamations, criminations, justifications, and a host of other *ations*. Take up a number of any of the French medical periodicals for the months of Sept. Oct. or Nov. and sure you will find the words "acarus scabiei," "acarus de la Gale," staring you half-a-dozen times on the wrapper page! We must frankly confess that, whenever we met with these ominous titles, we laid the number aside for the time, having no confidence in being able to give any thing like a "Periscopic review" of the multitudinous reports and descriptions. At length this labour has been done for us, by no less important functionaries than the members of the council of the Institute, and is embodied in the following report of Messrs. Blainville and Dumeril (two of the best comparative anatomists in Europe), on the various memoirs presented to the Academy by MM. Renucci, Beaude, and Sedillot, the three most zealous investigators.

Although several species of acarus were recognized by Aristotle and other ancient authors, and although the itch was certainly quite well known to the earliest physicians of Greece, it is somewhat singular that the first notice of the "acarus scabiei" is to be found in the writings of an Arabian physician, Avenzoar, who lived in the twelfth century. "There is (says he) a thing known by the name of 'soab,' which feeds and gnaws on the integuments, and when it becomes exposed, there escapes an animal so minute as to be almost imperceptible." In the Latin translation of Avenzoar's work, which was published at Venice in 1494, the author is made to use a more definite expression than he himself probably intended; for the Arabic word which signifies "a thing," "a substance," is translated "pedicelli parvunculi."

In 1657, Scaliger mentions the "acarus scabiei" in very distinct terms. "In describing (says he) the acarus of Aristotle, it is worthy of notice, that it is called by the Paduans "pedicello"—by the Tunisians "scirro," and by the Gasconese "brigant." It is of a globular form, and is so small that it can only with difficulty be recognized. It lodges under the epidermis, and causes a burning or stinging pain in the part; when extracted with a needle's point, and placed on the nail, it exhibits slight movements, especially if it be exposed to the rays of the sun—if squashed between two of the finger-nails, "on entend un petit bruit, et on en fait sortir une matière aqueuse."

Most of the physicians of those days received this description as true; and whenever they alluded to the itch, they invariably attributed its immediate cause to the existence of these acari. Aldrovandus is still more explicit and minute in his history:—After having described two species of acarus he adds—"recent authors have recognized a third species, called "scirro" or "pedicello;" it lodges under the integuments, creeping between the epidermis and the true skin, hollowing out sorts of sinuous galleries, and causing numerous vesicles on the surface. If one of these vesicles be opened, there escape animalculæ, which are so minute, that 'de tres bons yeux, et une vive lumière,' are quite necessary to see them." Nothing shews more satisfactorily the current belief of the existence of the itch insect, than the definition of the word 'pedicello,' given in the Della Crusca Dictionary, published in 1612; for the editors have quoted a verse from the works of a cotemporaneous poet, who said—"per fare nessuna ingiuria ai pedicelli, egli sempre ebbe cura di portare i guanti." It was the reading of this passage which induced Dr. Bononio, of

Venice, to write a memoir, descriptive of the appearances of the insect under the microscope, and to have drawings of it made, which were, indeed, the first ever published.

Muller has given a short notice, and an engraving of the acarus, in the Acta Eruditorum; and so, also, has Dr. Mead, in the Philosophical Transactions for 1702. Linnæus mentions, in his Fauna Suecica, the acarus scabiei, and designates it by the appellatives "humanus and subcutaneus;" he has stated that it is to be sought for, not in the pustule itself, but rather at its edges, under a certain spot where it lies concealed, the ova only being in the pustule. Geoffroy, in his Histoire des Insectes des Environs de Paris, 1762, repeats nearly the same description, and Morgagni tells us that he, on one occasion, extracted from a scabious vesicle several exceedingly minute whitish globules, which he found to be "de veritables acari." M. Latreille, in 1806, proposed to establish a new subgenus, under the name "sarcopte," for the reception of the acarus scabiei; but it is proper to mention, that he trusted to the correctness of the descriptions given by other authors, and had not satisfied himself by personal examinations.

In 1812, M. Gales, chief apothecary to the Hôpital St. Louis (into which all the "galeux" of Paris and its environs are admitted), availed himself of the extensive opportunities he possessed of determining the accuracy of preceding accounts; and he drew up a memoir descriptive of his observations. Many of his experiments, we are told, had been witnessed by several medical men, who all "avaient pu voir le ciron de la Gale." Not satisfied with this concurrent testimony, M. G. shewed, by an experiment made on himself before the commissioners, who had been appointed by the General Council of the Hospitals, that an acarus, placed on the skin of his arm, caused an eruption of pustules of genuine psora. From the date of this memoir until 1829, the descriptions and drawings of M. Gales were universally received as authentic, when, lo and behold, M. Raspail (*see* the Annales des Sciences d'Observations pour l'année 1829) detected that the figures of the acarus scabiei, which M. G. had published, represented, not this species of insect, but the common mite of cheese!! Once more, therefore, the very existence of the acarus scabiei was doubted, and most of the physicians in France were inclined to accede to M. Raspail's opinion—"that the parasitic animal of psoric pustules is only of occasional and accidental occurrence;" and we find that the Baron Alibert, in his treatise on Cutaneous Diseases, published in 1832, states—'que peutetre les acarus ne sont propres qu'à une espece de gale et â l'idiosyncrasie des sujets, et que, peutetre, ils ne paraissent que dans certaines années, et specialement dans certains climats.' This very qualified and hesitating statement of the chieftain of the "dermatophiles," very distinctly implies his great doubts if there be an acarus scabiei at all; the "peutetre" is a most unsatisfactory word, and, indeed, we find, on perusing M. Renucci's paper, that the Baron "manifestait de doutes sur la possibilité de trouver facilement l'animalcule." Such was the uncertainty of opinions last August, when M. Renucci undertook to demonstrate the much-disputed problem. It will be amusing to the English reader to follow the sketch he has published, in a late No. of the Revue Medicale. We are informed that he is a native of Corsica ("à laquelle je suis fier d'appartenir"), where the itch luxuriates most abundantly; "elle est une maladie endemique dans ce pays, dont elle est, je dirais, presque le fleau." The women of the place are very expert in extracting the acari, or, as they are called there, "pedicelli." M. R. watched them doing this operation, and at length acquired great dexterity himself. On going to Paris, he was surprised to find that the medical savans of the metropolis were really quite ignorant of what was so well known in his "chère patrie." He, therefore, undertook to enlighten them, and one of the patients in the Hôpital St. Louis was selected for the purpose of his demonstration:—"J'indiquai aux assistans le point ou devait se trouver l'acarus; je leur montrai les signes qui decelaient sa presence, apres quoi, j'en fis l'extraction, à l'aide d'une epingle." So complete was the exhibition, that the acarus moved "tres bien" along M. R.'s finger-nail, and every one present could distinctly see it with the naked eye. This experiment was repeated on another itchy patient in the hospital, and with equal success. The Baron was immediately freed from all the doubts which he had hitherto held, and, with praiseworthy speed, set himself at once to "dresser un proces-verbal de cette séance," which was forthwith signed by all the spectators. Many of the pupils speedily acquired as great cleverness at extracting the pedicelli as any of the Corsican ladies.

The only instruction which it is necessary to give to the young huntsman, is to look for, at the base of the vesicles (which must be entire, and not have been subjected to any treatment), the small grooves or furrows "qui se dirigent en differens sens;" these furrows are either directed towards the apex of the vesicles, or they pass round its base, and at other times they diverge from the base into the adjacent skin. At the extremities of these furrows, most remote from the vesicles, white spots may frequently be seen with the naked eye; at these spots, the epidermis will be found to be slightly elevated, and it is there that the hinder parts of the animalcula are lodged. In warm countries, a brownish-coloured spot may sometimes be perceived beside the white one, and this, we are told, indicates the position of the head of the acarus. Whenever either of these spots is seen at the extremity of the furrow, we may almost always calculate on finding an acarus beneath; and all that is then necessary for its extraction, is to insert the point of a needle and drag the enemy out. To unpractised eyes, it looks like a grain of flour, or a minute portion of the epidermis itself; if it is uninjured by the needle, and placed on the finger-nail, it will frequently, after a short time, exhibit signs of life, and speedily "ne tardera pas se mouvoir et à marcher avec assez de rapidité, pour qu'on soit obligé de le maintenir sur cette surface, de peur qu'il ne s'echappe." In conclusion, M. Renucci adds that the acarus is found frequently at the base of the vesicle, sometimes on its sides, but very rarely or almost never at its apex; and that it has been from ignorance of this circumstance, that the attempt to discover has so often failed. —*Journal Hebdom. et Rev. Med.*

---

### Medical Statistics of Algiers.

M. Maillot, the principal physician of the Military Hospital at Bon, in Africa, has published an abstract of the cases treated there during the month of June last, and has added some remarks on the prevalent disorders of the climate. At the beginning of the month, there were 237 cases in the hospital; during its progress, 935 cases were admitted, and, at the end of the month, 819 remained under treatment. The number of new cases exceeded considerably that of the corresponding period of the preceding year; and this increase was, no doubt, attributable to the earlier and greater heat of the present season. The prevailing malady was intermittent fever, which generally assumed the quotidian or the tertian forms. Of continued diseases, the most frequent were very acute "gastro-cephalitis," "irritations gastro-cephaliques febriles," and "colites dysenteriques." M. M. observed that the intermittents had become gradually more and more masked and complicated with cephalic or enteric disturbance, or with both, since the commencement of the hot weather. During the Spring months, they were distinctly marked, the paroxysms and intervals being well contrasted with each other; but, as the Spring and Summer approached, they assumed more and more of a remittent, and subsequently of a continued form. Out of 134 cases of ague, treated during the months of February, March, and April, there were not fewer than 56 cases, in which no traces of encephalic, pulmonic, or gastro-intestinal disease could be discovered; whereas, in 64 cases treated during the month of May, there were only five which could be called truly simple, or uncomplicated with some local mischief, either of the brain, or more frequently of the bowels.

In the following month, the same medical constitution prevailed; for, out of 162 cases of intermittent fever, only 18 were simple, and in all the rest there were distinct and well-marked affections of one or other of the three great visceral cavities. It is also worthy of notice, that, in addition to the greater frequency of such complications, the severity of these complications was found to increase, as the Summer heats advanced: the corresponding affections having more of a merely irritative character during the early spring months, and much more of a decided inflammatory character during the months of May and June. "C'est ce passage d'un degré leger d'irritation à un degré plus elevé, qui constitue le danger des affections du mois de Juin; c'est la congestion irritative, brusque, violente des principaux viscères qui fait passer ces fievres de l'etat de simplicité à un etat toujours grâve—souvent mortel; ce sont ces redoubtables phenomènes qui, portés au summum, leur valent le titre de fievres pernicieuses."

As might have been expected, the pulmonic affections were more prevalent during the early spring months, and the gastro-intestinal and the cephalic (these two latter being very generally associated) during the Summer ones. Whoever considers the facts now stated, cannot hesitate surely, to admit that the remittent and continued fevers of the hot season, in such a climate as that of the northern coast of Africa, are, in truth, intermittent fevers, complicated with a local mischief, which has the effect, sometimes, of merely obscuring the features of the original disease, and, at other times, of masking these altogether, so that they cannot be recognized.

Dr. M. observed that, almost invariably, every case of remittent fever, if seen at the earliest invasion, set in with one, two, or more paroxysms of genuine ague, the interval or intervals being well marked; and that, therefore, it was not for some time "que la reaction circulatoire ne tombant plus, il n'y avait plus d'intermittence." Moreover, the exacerbations were generally so violent, and so regular in their recurrence, even when the remittent type was once fairly established, that no doubt could be entertained of their essential or primitive nature; they were, in short, the paroxysms of an ague, which had lost its intervals of apyrexia. When the internal mischief is still more extensive and severe, the remissions of the feverish disturbance are less obvious, and the disease, no longer exhibiting those alterations of abatement and increase which characterise remittent, and more especially intermittent fevers, becomes what is called a continued fever. Even in epidemics of what are considered to be, essentially and primarily, continued fevers, it is not unfrequent to meet with cases which partake greatly of the character of remittents; but we do not mean to infer from this, that the two orders of fevers are invariably more or less associated with each other. Our present remarks are only intended to prove that, whenever a remittent fever assumes a continued type in its progress, we may be quite satisfied that some internal lesion has become more fixed and alarming.

As to the treatment of his patients, Dr. M. assures us that he has derived great benefit from having his mind constantly impressed with the preceding views:—He says, "c'est de l'idée qu'on se formera de ces intermittentes, de ces remittentes, et de ces fievres continues, se succedant tour à tour, se remplacant, se chassant, puis reparaissant, tournant, pour ainsi dire, dans le cercle annuel; c'est de la filiation, que l'on verra ou non entre ces maladies, si diverses en apparence, si identiques pour le fond, que dependra le choix d'un traitement vrai ou faux." In the uncomplicated intermittents, such as were generally observed in the early spring months, the lancet and the sulphate of quinine were almost invariably sufficient to effect a speedy cure. The depletion of blood, varying in amount, according to the constitution of the patient and the severity of the pyrexial symptoms, was always found to be a most useful preliminary. Whenever any of the great internal cavities was unusually oppressed, or otherwise disturbed, the application of a number of leeches near to the suffering part never failed to produce speedy relief. No sooner was the circulation calm and equalized by these means, than the use of the quinine was at once commenced, in large and frequently repeated doses. In the remittent, and also in the continued cases, the same line of treatment was pursued; but it required a nicer discrimination to determine the exact time at which the quinine was proper. As a general remark, however, we may state that, "toutes les fois qu'on a put saisir les moindres indices de remittence, on doit recourir au sulphate de quinine." The grand objects to be kept in view are to prevent, as early as possible, the return of the paroxysms, and to obviate or to remove congestions of blood in any of the great cavities. It was observed that every paroxysm added more and more to the risk of organic lesion, in some of the viscera, from a mere irritative congestion to inflammation, and subsequent disorganization of tissue. These are the mischiefs which are the source of the obstinate diarrhœas, the "colites," the dropsies, and the gorged livers and spleens, which are so common after epidemics of intermittent fevers.

In conclusion, we may repeat that the prevailing type of the fevers, at and in the neighbourhood of Algiers, is primarily and essentially intermittent; and that these fevers have a disposition to assume a remittent and continued form, in proportion as there is the tendency to visceral disease, whether this tendency arise from the constitution of the patient, or from any extraneous cause, such as the increase of the Summer heats, exposure to currents of cool air, &c. By keeping these principles steadily in view, the success which attended Dr. M.'s exertions was most gratifying. By bringing back, as it were, the remittent and continued fevers to their original type, viz. that of regular ague, in the manner we have already explained, and by the then bold use of the specific, most of the cases were led to a favourable issue.—*Journal Hebdomadaire.*

---

### Treatment of Amenorrhea, by irritation of the Mammæ.

'Docet doctrina sympathiæ non semper ad partem affectam remedia esse dirigenda' is an axiom of frequent practical interest to the medical philosopher in the treatment of disease. It is well known that certain parts, or organs, situated at a distance from each other, and having no direct bond of union, either by blood-vessels or nerves, are yet so intimately associated with each other in the performance of their functions, that whenever one of these becomes diseased, or in any way deviates from the state of health, the other almost immediately suffers, from the operation of that mysterious law, which has been called sympathy.

Nowhere are these phenomena so striking or so uniform in their occurrence, as in the case of the uterus and the mammæ. The affections indeed of the latter organs are seldom quite idiopathic, or independent of some uterine malady, and hence the paramount importance of attending to the state of the generative organs, whenever the mammæ are threatened with, or involved in disease. Hippocrates was well aware of this intimate sympathy, when he recommended the employment of dry cupping to arrest sanguineous discharges from the uterus, a practice, which may not unfrequently be resorted to with decided advantage. Within the last twelve months Dr. Rigby of London has published some cases in the Medical Gazette, establishing the utility of applying the infant to the breast to arrest puerperal menorrhagia.

Drs. Loudon and Patterson have the merit of having first extended the same therapeutic principle of mammary irritation to the treatment of amenorrhœa; the former by the persevering use of one or two leeches daily to the mammæ for several weeks, the latter by the application of sinapisms and other epispastics to these organs. The following two cases are corroborative of the correctness of their opinions.

A young woman, 21 years of age, fair, and of a very delicate constitution, consulted Dr. Mondiere, for a tumor in the right mammæ. It was of the size of a walnut, firm, even on the surface, and painful when pressed. She attributed it to a blow she received on the breast, three years before. The catamenia had not been seen for upwards of a year and a half.

During six months she had been treated for this amenorrhœa, by leeches to the vulva, and by various emmenagogue medicines; but without avail. Dr. M. advised the repeated application of leeches to the mammary swelling; but as this advice was not followed, he satisfied himself with prescribing frictions with iodine ointment, and the use of emollient cataplasm. This treatment was continued during two months, but without much advantage. Dr. M. therefore had recourse to 'un moyen tres peu connu;' viz. 'la succion souvent repetée du mamelon.' The gentleman, with whom the girl 'entretenait des relations' performed this delicate office, and he succeeded so well, we are told, that the mammæ very soon began to swell and be painful, and the nipple became red, extremely sensitive, and was in a state of almost constant erethism. So tender was the whole organ, that the suction required to be discontinued for some days. When the tenderness subsided, the suction was repeated with the same result as before. In the course of a few days, the catamenia made their appearance. At this time the breast was hard, swollen and painful, the nipple very irritable, and the whole organ so sensitive, that the gentlest pressure caused severe suffering. The tumor of the mammæ however did not receive any benefit from the treatment now explained.

In the second case, although there was not indeed any deficiency or irregularity of the menstrual flow, the effect of leeching the mammæ, on this discharge, was so striking, that the particulars deserve to be mentioned. A young married lady had a tumor in the left breast: it had existed for upwards of two years. During this period she had repeatedly applied to the tumor a few leeches at short intervals of time; and she had invariably observed that 'pendant l'application de ces annelides' the catamenia were not only much more abundant than they had been before, but also that they returned every third week. The tumor was at length extirpated by M. Lisfranc, and proved to be a fibro-serous cyst.

The third case was treated by Dr. M. in the manner recommended by Dr. Patterson (whose observations our readers will find at page 171 of the Med. Chir. Review, for January, 1834.)

M. R. aged 21, of a feeble constitution, had menstruated for the first time, when she was 19. During the first year, the catamenia were very sparing, irregular in their return and accompanied with head-ache, and general indisposition, which obliged her to keep her bed for two or three days. A sudden fright suppressed them altogether, and for upwards of six months, there was no appearance of any discharge. Her health had declined, and when she consulted Dr. M. in June last, she was in a state of chlorotic marasmus.

He recommended that just before the expected time of the catamenial discharge, a small sinapism be applied on the outer half of each mammæ, and kept on until some irritation was induced. On the day following the application, the menses appeared, and continued for 24 hours. The mammæ were for the space of two days, swollen and inflamed on the surface. At the next monthly epoch, the sinapisms were again used, and with still more decided advantage; for the catamenia this time continued for three days. In August and September, they returned regularly, although no means were used.—*Journal Hebdomadaire.*

---

### Treatment of Erectile Tumors (Aneurisms by Anastomosis) by Caustics.

Until the publication of a very valuable paper in the Medico-Chirurgical Transactions by Mr. Wardrop, surgeons entertained great fears of applying any form of caustic to these bloody swellings, and even in the present day, despite of many successful cases recorded by Mr. W. as well as by other English surgeons, there appears to be a strong prejudice against this mode of treatment on the Continent under any circumstances.

M. M. Boyer and Begin decidedly condemn it, and Professor Roux restricts its employment to the cases in which 'les tumeurs erectiles sont tout-à-fait superficielles, et assez peu developpées pour qu' on puisse les detruire par une seule cauterization;' he adds, 'mais dans toute autre circonstance, l'infidelité et incertitude de ce moyen, auquel d'ailleurs on n'a eu qu' assez rarement recours, doivent le faire proscrire entierement, d'autant plus que l'instrument tranchant, beaucoup plus sur, serait applicable à tous les cas ou l'on pourrait cauteriser.' M. M. Maunoir and Velpeau have recorded nearly the same sentiments.

Callisen in his System of Surgery, has these words; 'nævi parum prominentes caustico admoto consumuntur; sed majores nævi ferro exsecandi sunt;' but it is unquestionably to Mr. Wardrop, that the merit is due of having established the efficacy of the treatment by caustics, in certain cases of nævus. The first case in which he employed it, was that of an infant 13 months old; the nævus was situated on the middle of the forehead, and was not much larger in its disc than a sixpence. A rod of the kali purum was rubbed on the centre of the nævus, until the skin became of a deep brown colour. The eschar gradually separated, and a complete cure was effected in three weeks.

Another nævus of equal dimensions, on the cheek, was treated in the same manner, and with the same success. In a third case, the tumor was of much larger size, and affected the cheek and lips. Mr. Wardrop applied the caustic to different parts of the nævus successively, and effectually destroyed the whole disease in the course of a month. This case had been considered of very unfavorable prognosis by many surgeons, who had seen it, before the treatment was commenced. The fourth example was still more remarkable.

A child, two years of age, had a large pulsating erectile tumor, on the front of the thorax, a little to the left side of the sternum. The application of the caustic required to be very often repeated, in order that the whole mass might be effectually destroyed. This treatment was persevered in for nearly five months; the suppuration was at one time very profuse, but this could be always moderated by applying the balsam of Peru; and fortunately no hæmorrhage occurred at any period. The cicatrix many months after the cure was complete was found to be firm although highly vascular: this vascularity however appeared to be rather venous than arterial.

Dr. Lee has followed the example of Mr. Wardrop, in two or three cases of nævus; in one, the tumor of the size of a small egg, was situated on the fronto-parietal space of a young infant; it pulsated strongly (the pulsations were partly attributable to the movements of the brain), and more than once the skin had broken, and a quantity of blood been discharged. Dr. L. fearing that the swelling might be connected with the dura mater, used the caustic with great caution; the applications were made, at short intervals, for many months: but at length a complete cure was obtained, and this was the more satisfactory, as the situation of the nævus had precluded other modes of treatment. In a second case, the cure was much more speedy, in consequence of the caustic having been more freely used; the swelling was originally of the size of a walnut, and occupied the vertex of the head. The cicatrix was white, smooth, and shining.

Mr. Higginbottom, of Nottingham, has published in the Medical Gazette two cases of congenital nævus, cured by the application of the potassa fusa. In the first of these, the nævus, of the size of a walnut, and situated on the left side of the inferior maxilla, was gradually becoming larger and larger; one free application of the caustic, according to the method proposed by Mr. Wardrop, was sufficient for the removal of the disease. In the other case, the nævus occupied the left wing of the nose; five applications of the caustic were necessary for its removal—the cicatrix which remained was scarcely perceptible. Mr. H. has succeeded in other two cases, the particulars of which have not, we believe, been yet published.

It will, no doubt, be remarked by the reader of the preceding observations, that all the cases now recorded occurred in very young children; to them, Mr. Wardrop's treatment is especially well adapted, as the application produces but little pain, and a very trifling constitutional disturbance; the application may be made while the child is asleep, and will often not even awake it. The chief objection is the slowness of the progress, and the tedious delay which is necessary in some cases. It is to be remembered, however, that the cauterization of the whole diseased surface is not always requisite; for even a single application to one point of the nævus has succeeded in numerous instances; and Mr. Higginbottom alludes to a case, which occurred in his practice, where he did not see his patient for several weeks after he had applied the caustic for the first time; and, when the child was brought to him again, he found that the disease was very rapidly subsiding: eventually it completely disappeared, although no further medication was employed.

A most interesting case of an erectile tumor, in the genital organs of a young woman, was recently admitted into the Hôpital Necker, at Paris. The tumor was situated immediately beneath the urethra, extending upwards into the vagina for some distance—when pressed with the finger, the patient felt a good deal of pain. M. Laugier applied the potassa fusa to the centre of the swelling; a slight oozing of blood took place. Without waiting for the separation of the eschars, M. L. repeated the cauterization twice; the tumor was soon reduced to half its original size, and felt to the finger much firmer and denser than it was at first. It was now discovered that this girl was pregnant; and, unfortunately, she left the hospital before the cure was completed, although no doubt could be entertained of the final success of the treatment. In concluding his memoir, M. Tarral alludes to the use of the caustic in some cases of hæmorrhoids, and also of varicose veins of the legs; and he is inclined to prefer this mode of treatment, in varicocele, to the ingenious plan recently proposed by M. Breschet, of obliterating the enlarged veins by means of pressure. The authority of the distinguished Prussian surgeon Baron Graefe, may be adduced in favour of the treatment of some nævi by cauterization; but, instead of using the potassa fusa, he employs the heated iron.—*Archives Generales.*

---

### PARTIAL OSSIFICATION OF THE MUSCLES OF THE SHOULDER, IN YOUNG MILITARY RECRUITS.

Although every surgeon of an infantry regiment must have witnessed many cases of a partial ossification of the deltoid of the left shoulder (in consequence of the pressure of the musket on this part) in soldiers, it is rather singular that, with the exception of Kuhn, of Potsdam, and Richter, of Dusseldorf, no author has published any account of this bony transformation. Dr. Hasse, in a paper recently published in the Transactions of the Physico-Medical Society at Kœnigsburg, states that, in examining 600 recruits, who had entered the service within the preceding twelvemonths, he met with eighteen cases of this affection; the size of the ossific deposit varying from that of a pea to that of a goose's egg, and its consistence from that of a stiff jelly to that of perfect bone. The earliest sign of the disease is an inflammatory swelling, red on the surface and painful, an inch or two below the coracoid process of the scapula, and exactly at the point where the stock of the musket rests.

The soldier usually washes the part with a spirituous wash, and this has the effect of dispersing, indeed, the superficial inflammation, but very often of driving it more inwardly, till it penetrates to the deepest muscular layers of the deltoid, biceps, and pectoralis major, and assumes that peculiar action which occasions a deposition of osseous matter in the muscular tissue. The stages of this process may be considered as three: in the first, a small tubercle, like a gland, may be felt, moveable under the skin—in the second, this tubercle becomes larger, and acquires a cartilaginous consistence; and lastly, after a lapse of two or three months, when the degeneration has become complete, the deposit has all the firmness and hardness of bone. It will be readily conceived that the degree of lameness of the extremity will vary in different cases, according to the extent of the morbid change, and the exact position where it has taken place.

Pieces of bone, from three to five inches long, and one or two in thickness, and weighing from two to eight drachms, have been extracted; they are usually of an irregular shape, broader above than below, and rough on their surface. Kuhn once found a foramen for the passage of a bloodvessel through a piece which he had excised. If the transformation into bone has not been complete, we have often a most distinct illustration of the different stages or phases of the ossific process, from the change of the muscular fibres into a cartilaginous silvery tissue, and then, from this, into a substance which is porous, and exhibits osseous spiculæ at different points. When once the ossification is complete, "on trouve partout, meme apres une maceration anterieure, un veritable tissu osseux, garni de petites cellules, et recouvert en haut d'une couche en partie tendineuse, et en bas d'une couche musculaire." The extirpation of these bony masses is sometimes very troublesome and tedious, in consequence of their adhering closely and very intimately with the surrounding muscular substance.—*Observateur Med. Belge.*

---

### Poisoning with Corrosive Sublimate.

The three children, whose ages were 7, 3, and 2 years, of Mad. Nelissen, during their recovery from the measles, were ordered by the attending physician to have some powders of the protochloruret of mercury (calomel); the eldest child was to take eighteen grains—the second twelve grains, and the youngest six grains. The mother, having previously mixed each powder with some sugar, and dissolved it in a spoonful of water, administered to each of her children their respective doses. No sooner had the eldest child swallowed rather more than one-half of his medicine, than he was seized with retching and vomiting; but, as these were supposed to be the effect only of the repugnance of the child to taking physic, the remainder of the dose was immediately given. The distress of the stomach became speedily aggravated, and was accompanied with convulsive movements of the body, and with copious alvine dejections. The "pharmacien" was summoned two hours after the accident, and he directed that the child should drink a quantity of milk, with the whites of two eggs beaten up with it. In the course of another hour, the little patient, after having suffered extreme agony, succeeded by a state of exhaustion, and by occasional convulsions, expired.

The second child, immediately upon swallowing his dose of twelve grains, was attacked with violent vomitings of a bloody fluid, and these weie speedily followed by purging, and then by a tendency to stupor, which was every now and then interrupted by excruciating pain in the bowels, and by desire for stool. The youngest was affected in a similar manner, and died in eleven hours after the exhibition of the medicine.

These melancholy accidents could not fail to suggest the probability of an acrid poison having been swallowed by each of the children; and the suspicion was increased by the circumstance of the tin spoon, in which the powders had been dissolved, having become of a brownish-black colour. On applying to the chemist, at whose shop the prescription (which was found to be written most legibly and correctly*), it was at once discovered, that his pupil had used the corrosive sublimate instead of calomel. The sufferings of the second child were protracted until the twenty-third day after the accident, at which time he died, with all the symptoms of an obstinate enteritis. The following account of the post-mortem examination of the bodies of the other two children had been drawn up by Drs. Ollivier and Barruel, who were charged by the "Procureur du Roi" to make an official report.

The corpses had none of the usual rigidity of death: the limbs were flaccid—the bellies were enormously tumefied—the epithelium of the mouth, pharynx, and œsophagus was, in several places, soft, white, and could be easily detached by the handle of the scalpel. The mucous surface of the stomach and bowels exhibited patches of acute inflammation, and here and there of partial erosion; at these places, its colour was of a deep brown, or of an eschar-like hue. On the inner surface of the left ventricle of the heart, in the elder child, there were observed two patches of distinct ecchymosis, caused by the effusion of blood between the investing serous membrane and the muscular tissue. In the younger child, also, a similar appearance, but less distinctly marked, was found. [It is worthy of notice that Orfila, in his "Toxicologie Generale," has alluded to his having seen this phenomenon in several of his experiments on animals.—REV.]

*Chemical Examination.* The spoon in which the powders had been given, was first examined. On being immersed into a measured quantity of pure distilled water, the brown-coloured coating, with which it was lined, gradually separated, and formed a sediment at the bottom of the glass. The water was decanted off; and, being treated with the "acide hydro-sulphurique" (sulphuretted hydrogen), it yielded a dirty, yellow-coloured precipitate, insoluble in some drops of ammonia. An excess of nitric acid was added to the water, and the mixture boiled for one hour. On applying the nitrate of silver, a white, curdly precipitate, insoluble in nitric acid, but readily soluble in ammonia, was immediately formed. This experiment proved that the substance, which had been held in solution by the water, was a metallic chloruret.

The brown deposit from the spoon, being carefully dried, and examined with a magnifying-glass, was found to exhibit numerous spiculæ of a silvery brilliancy; and, when it was triturated with a glass rod, quicksilver-like globules could be at once perceived. It was then introduced into a test-tube, the open end of which was, by means of the flame from a blow-pipe, drawn out into a capillary bore, and a gradual heat applied to the larger end, where the brown powder was, with the precaution of inclining somewhat the tube to the horizontal position. The powder was speedily volatilized, and the cool surface of the tube became coated with numerous resplendent metallic globules, evidently those of mercury, and which were proved to be so by dropping them on a plate of gold, on which they immediately formed a white amalgam. The tube was broken, and the residue of the brown powder, being collected, was treated with pure nitric acid, which readily dissolved it with considerable effervescence. This solution was divided into three portions, and these three portions were respectively treated with a solution of sulphate of soda, with the "acide hydro-sulphurique," and with the chloruret of potass: the first yielded a white precipitate—the second a black one, and the third a yellow one. These experiments proved, that the residue of the brown powder in the tube consisted of lead, which doubtless had been derived from the pewter spoon.

To render the examination of the spoon still more conclusive, the synthetic method was resorted to after the

* We suppose that the term "protochloruret" had been used—a most dangerous refinement.—REV.

analytic. A few grains of the corrosive sublimate, being mixed with sugar, were dissolved in water held in the spoon, after it had been well scoured; its surface became immediately blackened, and when allowed to dry, and immersed in distilled water, the same appearances as those reported above, were in every respect repeated.

We shall now proceed to describe the analytical examination of the contents of the stomach and bowels. It has been already noticed, that a considerable quantity of milk, and the whites of several eggs, had been administered to the eldest child by the druggist, who had been summoned to her relief. Orfila recommends the use of these two articles, as the most powerful antidotes against corrosive sublimate by depriving it of a portion of its chlorine, and thus converting it from the deuto into the proto-chloruret, or calomel. It became, therefore, a question of much interest to determine, whether this counteracting effect had really taken place in the stomach. But, first of all, it was necessary to examine the fluid which was found in the stomach, and the water used in cleansing it: these having been filtered, so as to separate all the solid matters mixed with them, were treated with a concentrated solution of sulphuretted hydrogen; but they exhibited no change of colour, nor did they yield any precipitate. From the result of this experiment, we inferred that the death of the child had been caused by a metallic poison; the salt was certainly not held in solution, unless, indeed, it had been all discharged by vomiting; and, in the present case, the discharges had not been preserved. The next step was to examine the solid contents; these consisted almost entirely of a soft, coagulated mass—no doubt the remains of the milk and eggs which had been swallowed.

This pulpy mass was washed with pure distilled water, and the water was tested with the sulphuretted hydrogen; but no change was induced. It was then well mixed and triturated with a solution of potassa, for the purpose of dissolving the caseous matter, and, at the same time, of precipitating the black oxide of mercury, if any calomel was really present. The solution was quickly effected; but, instead of a black precipitation, a number of yellowish flocculi were formed—on the surface of the solution, an oily matter, the buttery part of the milk, floated. The yellow flocculi, being mixed with a small quantity of distilled water, was subjected for several hours to a stream of chlorine gas: this at first caused a further separation of white flocculi; but, upon the process being continued until the water became strongly acid, all the flocculi were dissolved. The fluid was then slowly evaporated to one-half its quantity, and into it a thin plate of gold, surrounded with a spiral plate of tin, was introduced. A perceptible effervescence speedily took place, and the gold plate became whitened on its surface, in consequence of an amalgam having been formed upon it. To prove that this was really the case, the plate, previously separated from the tin one which encircled it, was put into a test-tube; the open end of this was then drawn out into a capillary bore, and the large end heated to redness. On cooling, the drawn-out extremity of the tube was found coated with numerous distinct globules of quicksilver, and the gold remained behind pure. The tin plate was treated in the same manner, and yielded the same satisfactory results.

It is unnecessary to detail the results of every step in the examination; but it is of high importance to keep in mind the proper method of conducting such an enquiry;—first, the careful washing of the contents of the stomach, and of the bowel itself, with boiling distilled water, adding, from time to time, small quantities of caustic potass; then the treating the fluid with an excess of hydrochloric acid; and, lastly, the filtering it, and introducing into the filtered fluid a gold plate, as has been already mentioned.

The result of the preceding investigation is highly instructive, as teaching us how an albuminous matter, such as milk and the whites of eggs, acts in obviating the effects of the corrosive sublimate. We do not mean to deny that it may sometimes operate, by converting the deuto into the proto-chloruret; but certainly this was not the way in the present case: and it seems to have acted rather by enveloping the acrid particles, and thus preventing it from becoming dissolved, than by causing any decomposition of its ingredients; else, how should some portion of it not have been dissolved in the distilled water, in which the curdly contents of the stomach of the eldest child had remained for several days?

We have no hesitation in impressing upon our readers the necessity of *immediately* resorting to copious draughts of milk and of whites of eggs, mixed together, whenever they are called to a case of poisoning with the corrosive sublimate.—*Archives Generales.*

---

### Observations on some Subjects of Medical Jurisprudence.

It was our intention to have embodied, in a general review, the following extracts from M. Trebuchet's late work on "la Jurisprudence de la Medicine, &c." published last year in Paris; but, in the progress of our labours, we soon found that the work was not well suited to our purpose, from the extreme multiplicity of its details, many of which are most vague and uninteresting, and from the inextricable confusion of their arrangement. As detailed observations, they will amuse and instruct the mass of our readers.

### Death from Hanging.

The memorable case of the unfortunate Calas, who was condemned by the Parliament of Toulouse, in 1761, to the torture of the wheel, for the imputed crime of having murdered his own son, is one, among many, of the shuddering examples of error into which medical witnesses may fall. The son was found suspended from the beam over a door, which led from the shop of his father into an adjoining apartment. On the following day, the corpse (which had most improperly been conveyed to a hospital) was examined by a physician and surgeon, who, without examining the cord, the fatal instrument of death, or even the room in which the catastrophe had taken place, decided, ' purement et simplement," that the young man had been forcibly strangled. Four years afterwards, the innocence of the father was publicly admitted "par le Grand Conseil," and his memory "rehabilitée par un jugement definitif." There are, indeed, few cases of violent death, in which it is more difficult to determine the question whether the act has been suicidal, or been perpetrated by another, than those wherein the bodies of the victims are found suspended. In a late No. of the Gazette Medicale, is an instructive example of the perplexing circumstances which such cases may present.

A man who had become deranged some time before, was one morning found suspended from a door of a privy in the neighbourhood. The rope had been secured to the upper hinge of a door, which was scarcely five feet above the floor; whereas the body considerably exceeded this height, and the man must, therefore, have rested with the heels and toes on the ground, had not the limbs been voluntarily drawn up. Now, in this case, there was not even the shadow of suspicion that the fatal act was not strictly suicidal. In truth, all cases of death from hanging carry with them the presumption that they have been perpetrated by the individuals themselves, seeing that this is by no means a very easily effected method of murder, unless it has been committed when the victim was asleep, or otherwise stupefied. Still it is quite necessary that the medical jurist examine minutely every particular of the case, as it is possible that strangulation may have been perpetrated in some other method, and the body of the victim have been afterwards suspended, for the purpose of misleading the enquiries of justice. Some authors have very properly urged the importance of examining the state of the finger-nails, and, indeed, of every part of the corpse, to ascertain whether there are signs left of violent struggles having been made by the sufferer previous to death ; but it is to be remembered, that the presence of such signs is necessarily inconclusive. On the other hand, the total absence of them is strong presumptive proof of the crime having been voluntary, unless there is reason to suspect that murder had been committed before the body was hung. An instance of such a "meurtre rusé" occurred last year in Germany : the unhappy criminal confessed that she and her paramour had first strangled her husband, and then hung the body from a rafter of the roof.

It will no doubt occur to every intelligent witness, to endeavour to ascertain in a case of death from hanging, whether the victim could alone and by his own efforts have suspended himself. Supposing the body be found hanging at a considerable height above the floor, it would naturally be enquired whether any table, chair, &c. on which he might have mounted, was at, or near the spot. Professor Remer of Breslau, alludes to a case of a young man being found hanging from the slender branch of a tree, at a considerable height above the ground. No evidence could be adduced, to prove that the crime had been committed by another; and the only explanation which could be offered, was by supposing that the unhappy man had bent down the branch, secured the rope to it, and that after he had put it round his neck, the elasticity of the branch had been sufficient to elevate and retain his body from the ground. The preceding considerations must satisfy medical men of the necessity of being cautious in pronouncing any very positive opinions, even where the circumstantial evidence bears strongly against the accused. The affirmation of M. Trebuchet, that he has collected the particulars of fourteen cases of suspension, in which the corpse rested on the ground, either with the feet, knees, or even with the nates (and under these circumstances we need not say that the death, if suicidal, must have been most determinedly voluntary), is worthy of the most serious attention, and proves how incorrect may be the opinion of those medical jurists, who have too precipitately concluded that strangulation, if perpetrated otherwise than by hanging, must have been the act of another, and not possibly of the victim himself. The resolute firmness of some suicides is indeed often most remarkable. A woman, mother of a large family, left the room, where her children were playing, into an adjoining one, and there suspended herself from the ceiling. Her eldest daughter happening to enter almost immediately afterwards, saved her by pulling at her garments so forcibly as "rompre le lien." The miserable woman, the cord all this time about her neck, put the child out of the room, secured the door, and succeeded this time in perpetrating the horrid act of self murder.

### Difficulty of Determining the Cause of Death in some Cases.

It does not belong to our present enquiries to enter upon the physiology of death from strangulation ; but the subject naturally leads us to dwell upon those cases of sudden natural death, wherein the corpse of the deceased may exhibit appearances very similar to those induced by strangling.

A fatal attack of apoplexy is an apt illustration of this remark. It is strikingly exemplified by the following details.

The corpse of a man of the name of Courbon was found in a ditch near the village of Dunieres, in the department of the Haute Loire. It was proved that he had visited several cabarets on the preceding evening, and had drank freely at each, so that when he left the last one, he was considerably intoxicated. The ditch in which the body was found, lay near the road which led from the public house to his own home.

Some vague suspicions against three brothers-in-law, who had been in company with the deceased at one of the cabarets, speedily got wind, and became the topic of common talk, and as our author expresses it ' une sorte de bruit populaire, semé par la calomnie et accueilli par une sotte credulité eclata contre eux.' They were arrested, tried and condemned; the leading evidence against them was that of a man, who had occupied a chamber in the inn, immediately adjoining to that in which the three accused were lodging, and who deposed that he had overheard them talking of the crime, which they had just committed. The jury found them guilty of the murder; but admitted that it had not been premeditated, and had been perpetrated under the influence of intoxication. One of the accused, who was believed to have been less concerned than the other two was condemned to imprisonment for twelve months, and the other two to be branded and sent to the galleys for life. The sentence had been already carried so far into execution, that the prisoners had been sent off to the 'bagne' at Toulon, when some circumstances came to light, which caused the evidence of the forementioned witness to be suspected, and it was now his turn to be arrested, and his testimony re-examined. Distinct proof was brought forward that it was almost impossible that a conversation held in the one room could be heard in the other, and moreover, that this wretched witness had in truth not been in the room at the very time when he had alleged having overheard the talking of the brothers. The case was clearly established, and the punishment which he had caused to be inflicted on two of his fellow beings, was now drawn down upon himself. We need not copy our French brethren's raptures on the "rares talens" of the advocates, the "debut si touchant et solennel," and the admirable heroisme of the prisoners' wives, one of whom, we are told, rivalled in her magnanimity "la femme forte dont parlent les livres saints." The inference in short from the whole affair was, that Courbon, in a state of intoxication had fallen accidentally into a ditch, and there died a natural death. This conclusion was amply confirmed by the report of the dissection; the encephalon presented all the usual signs of an apoplectic seizure. (Our readers will probably agree with us, that the details of the case are little creditable to the sagacity and patient enquiry of French judges and juries.) As illustrative of errors which have been, and therefore may be committed by incautious medical men, in their evidence on cases of suspected murder, we shall glance at another case of recent occurrence.

A young man was found dead in his bed-chamber, with three distinct gashes on the front of the neck. The physician who was first called, had stamped in the blood with which the floor was deluged, and had then walked into an adjoining room, passing and repassing several times, and had thus left a number of bloody foot-prints on the floor. No notice was taken at the time of this, but on the following day, when the examination was resumed, the circumstance of the foot prints was particularly attended to, and excited the suspicion that the young man had been murdered. Forthwith a person was arrested, and would, we are told, have "infailliblement subi les rigueurs inevitables d'une instruction criminelle" had not M. Marc, one of the leading physicians in Paris been called in to examine all the particulars of the case. So determined appears to have been the suicidal deed, that, as mentioned above, the deceased had made three distinct deep incisions across the throat. The lowest was about two inches above the sternum, and had divided the trachea, œsophagus, the carotid artery and the jugular veins almost completely across, and had even reached the anterior ligament of the cervical vertebræ; the other two incisions were higher up, but had not penetrated deep enough to wound these important organs.

Many similar cases might be adduced to prove that self-murderers have frequently failed in their dreadful design, at the first and second attempt, and that it has been only by repeated incisions made either in the same, or in different places, that they have at length succeeded.

### Monomania, inducing to Murder.

Our author, who is by no means remarkable for the "lucidus ordo" of his arrangement, introduces an episode on that form of monomania, in which the mind seems to be overpowered with the horrid propensity to commit murder. This is certainly a subject of the most puzzling difficulty, and deserves the patient examination not only of medical, but of legal jurists. It is only of late years that much attention has been paid to it, and although we may not be inclined to accede to all the speculations of the phrenologists (for it has been most assuredly to them that the merit belongs of first boldly canvassing the subject), no friend of justice and humanity can be willing to refuse a willing ear to the narration of authentic details.

A peasant, 28 years of age, born of healthy parents, had been affected since his boyhood with attacks of epilepsy. Within the last two years, his malady had experienced a melancholy change; for instead of the paroxysm of the convulsive order, this unfortunate man "se trouvait attaqué d'un penchant à commettre un meurtre;" he was so conscious of the approaches of this horrid feeling, for some hours previous to its invasion, that from the moment of its birth, he besought his attendants to confine him, and to remove every weapon of destruction from his reach; his own words were "Lorsqu'il me prend, il faut que je tue, que j'etrangle, ne futce qu'un enfant." "Ma mere, sauvetoi; ou il faut que je t'etouffe." Before the invasion of the fit, he felt as if oppressed with drowsiness, without however being able to sleep, and his limbs were affected with slight convulsive movements; during its domination, he confessed that he was all the time well aware of the atrocity of his wishes; and yet could not master or restrain them. A case in all respects similar to the preceding occurred in the person of a gentleman of good education, and of naturally mild disposition who was recently in one of the "maisons de santé," near Paris; he left several letters in which he had attempted to describe his state of mind before and during the demoniac possessions.

A volume might be filled with analogous examples. Awful indeed and revolting is the subject, but not the less necessary to be studied by the philosopher of human nature. Poets may feign, and romancers may conjure up all the most hideous devices of infernal agents, but alas! the historian of crime, or shall we rather say of insanity, can appal the mind with tales of darker horror. In the annals of 1726, we read that Richard and his wife Bridget Smith murdered their only infant in its cradle, and then hung themselves to the posts of their bed. A letter was found, in which was written—"We believe that God will forgive us—we leave the world, because we are wretched, and without support, and we have murdered our child, to save it from the like misery." (It is unnecessary to allude to the still more frantic tragedy, which within the last few months was perpetrated at Pentonville.)

### Detection of a Murder, by examining the Skeleton Twelve Years after Death.

The annals of last year detail a case, which is one of the most memorable in many respects that is on record. In September 1821, a widow of the name of Houet, disappeared from her residence, and no traces could be discovered at the time of what had become of her. Twelve years afterwards, viz. in April 1833, in consequence of some suspicious circumstances having come to light, the officers of justice, accompanied by M.M. Chevalier and Boys de Loury, were ordered to repair to the house No. 81, in the Rue de Vaugirard, and to examine all the trenches in the garden belonging to it. After much careful search, some bones were found, and these were at once recognized to be human. The earth was cautiously removed all round, in order that the position of the skeleton might not be disturbed. A quantity of quick lime was found mixed with the soil. When all this was dug away, the position of the different bones was easily perceived; and the opinion which M. Loury formed, was that the corpse must have fallen, or been thrown into the trench head foremost, for all the bones of the lower extremity were at a less depth than those of the cranium and shoulders. All the soft parts of the head had disappeared, except a few fragments of skin, which were recognizable only after having been well washed. The muscles of the chest, of the spine, and on some parts of the hips and thighs had become changed into masses of a black, or greenish brown colour, in which no traces of their original texture could be discovered. In other parts the muscles had assumed a greasy or a soapy character. The ribs were held together by a dry leathery membrane. The abdominal viscera were converted into a homogeneous tarry matter, which lay in the hollow of the pelvis. The brain was shrivelled to a small mass of a waxy consistence, and of a green hue. The tendons and aponeuroses of the shoulder, and some of the other joints were still remaining. Round the os hyoides and cervical vertebræ was found a thick string, or cord, having several turns upon itself. This was a most suspicious circumstance, and naturally suggested the idea of the unfortunate woman having been strangled. Several tufts of hair of a brownish colour, verging to grey were adhering to the cranium—a gold ring also was found in the trench. By sifting the earth, almost every small bone, and even every nail were at length discovered. The men (grave diggers from the Père la Chaise), who were employed in digging out the earth, gave it as their opinion, that the pit had been made by persons unaccustomed to this description of labour; the walls of it were irregular and sloping inwards, so that the dimensions were much more contracted below than above. The bones having been all carefully cleansed and arranged, the next step in the enquiry was to ascertain, whether they all belonged to the same skeleton; whether the skeleton was that of a male, or of a female; what was the probable age of the deceased, from the appearances of the bones, teeth and hair; how long the body had been in the earth, and so forth. As to the first of these interrogatories, there could be no doubt, that all the bones appertained to one skeleton; almost every individual bone, with the exception of a few of the carpal and phalanges of the fingers were found. The general aspect of the bones, and more especially the configuration of the pelvic bones, abundantly proved that the skeleton was a female one. The appearance of the cranial sutures, the worn-down surfaces of the teeth, the changes of the alveolar processes, the "affaissement" of the anterior parts of the bodies of dorsal vertebræ, the concretion of the cornua of the os hyoides with its body, and the circumstance of some of the hair being grey, or nearly white, all these phenomena indicated that the individual was considerably advanced in life, and probably 65 years old, or thereabouts. The length of the skeleton was determined to be nearly five feet. Such are the data for solving some of the questions, and we come now to the consideration of the inferences to be drawn, from the presence of the cord round the cervical vertebræ. The cord was about a quarter of an inch in thickness; there were six turns or coils "superposés, et affectant une direction presque horizontale; avec une legere obliquité de haut en bas, et d'avant en arriere." The knot or hitch was not found; it had crumbled to pieces. "La position de la corde etablit clairement que la personne a été etranglée sans suspension. Car, dans ce dernier cas, l'obliquité serait de bas en haut, et d'avant en arriere, ou horizontalement, ce qui arrive plus rarement." In reference to the probable period of inhumation, we have to consider not only the appearances of the skeleton itself, but also the nature and condition of the earth, &c. In the present case, the soil was of a sandy texture, and therefore not very favorable to speedy putrefaction, and moreover the corpse had been surrounded with a vault of lime, which by desiccating the softer parts, must have contributed to retard the decay still longer. We may reasonably suppose that the body had been in the earth for a considerable number of years, probably eight or ten.

The following, therefore, are the conclusions which we have deduced from the examination.

1. That the bones belong to a human skeleton.

2. That the skeleton is that of a female.

3. That the woman was probably from 60 to 70 years of age.

4. That her height was nearly, but not quite 5 feet.

5. That her hair, which had probably been fair brown in youth, was at the time of her death white and very short.

6. That her hands were very small.

7. That the bones had experienced no lesion during life.

8. That this woman had died from strangulation, and that this was in all probability the act of others.

9. That the corpse had been buried for several years.

(Signed)
Orfila, Marc & Boys de Loury,

*Annales d'Hygiene.*

### Case of Poisoning, detected seven Years after Death.

The body had been interred in ground which was rather elevated, and the soil of which would rapidly absorb any moisture. The coffin, when exposed, was found entire, but very fragile, and so dry, that its inner surface "n'etait pas meme tachetée par l'humidité." The corpse was entire; the head, trunk, muscles, &c. retained their natural position; the thoracic and abdominal viscera were completely disorganised; the only traces of them being a soft, brownish matter resting on the sides of the spinal column. It was in this *matter* that M. M. Ozanam and Ide discovered the presence of arsenic by the following processes. The matter was boiled in repeated quantities of distilled water as long as this (the water) was in the least degree discoloured. The different decoctions were then mixed together, and the whole evaporated to a dry extract, which was re-dissolved in boiling distilled water; but as this solution was still of a deep colour, it was again evaporated to dryness, and the residue was deflagrated in a porcelain vessel, with nitrate of potass; the saline mass thus obtained was dissolved in water and treated with nitric acid, and then with a solution of pure potass. The presence of the arsenious acid was most satisfactorily detected by applying the usual well-known tests to different portions of the solution obtained in the above method. —*Orfila, Gazette de Santé.*

### Anecdotes respecting Impotence, Affiliation, &c.

Having dismissed the division of his subject which refers to "medecine legale judiciaire *criminelle*," M. Trebuchet proceeds to the examination of "medecine legale judiciare *civile*," a no less important theme, than the former. It teaches, says he, the duties of medical men, when summoned before the civil courts, and instructs them how to give their testimony and opinions on cases of alleged insanity, whether the enquiry has been instituted for the purpose of depriving an individual of his civil rights and the management of his own property, or for the purpose of setting aside a marriage, will, or other responsible act; it treats also of alleged hermaphrodism, precocious puberty (for this when unequivocally proved may be deemed a sufficient reason for dispensing with the "art. 144 du code civil," which appoints that a male, before he has completed his 18th year, and a female under 15 years of age cannot contract marriage); of alleged, or concealed pregnancy; of alleged premature, or protracted gestation; of impotency and so forth. Without any regard to the priority of the other subjects, our author flies off at a tangent, on the mere mention of "impuissance," and not satisfied with detailing the modern usages in reference to this calamity, he glances retrospectively at the times, when, the virility or feminility of one of the parties being disputed, "le mariage etait consommé en presence de temoins nommés à cet effet par le tribunal." These interesting exhibitions were known by the name of "congrès." France, Italy,

England, and many other countries, had their "congrés;" and, we are told, "l'Angleterre, surtout, nous en fournit un exemple remarquable dans la procédure qui fut suivie sous le roi, Jacques 1er, dans l'instance de divorce que la Comtesse d'Essex intenta contre son mari." The "affaire" of the Marquis de Langey, who, although "jouissant de toute la plenitude de ses facultés physiques," was condemned to pass through the ordeal of the "congrés," was the occasion of the French Parliament abolishing, in 1677, this most strange and indecent proof. From that period, the plea of sexual impotence has not been sustained, unless it is "visible et absolue;" and, even under these circumstances, the courts are ever unwilling to sustain the allegation.

With respect to the laws of the French civil code, which refer to the subject of disputed paternity, we observe that, although the article 312 ordains, that "a child born in wedlock, shall be considered to be the offspring of the husband," yet the subsequent sections modify the rigour of this enactment so far, that the husband is permitted to disavow the child, if he can satisfactorily prove that he was, "soit par eloignement, soit par l'effet de quelque accident, dans l'impossibilité physique de cohabiter avec sa femme," from the 300th to the 180th day before the birth of the child, provided always the child is "viable," or capable of living: the "non-viabilité" arising from monstrosity precludes any appeal to the provisions of the article quoted above.

According to the art. 315, the legitimacy of a child born 300 days after the absence or death of the putative father, "pourra etre contestée;" and, again, a child "n'est pas legalement viable, lorsqu'il est né avant le cent quatre-vingtieme jour de sa conception, c'est à dire, avant six mois." As a consequence of the former of these enactments, the 101st art. of the penal code appoints a heavy penalty on an "officier de l'etat civil," who permits the marriage of a woman, once wedded, within ten complete months after the dissolution of the first marriage; for, otherwise, she might be pregnant at the very time of her second contract. From marriages, we naturally proceed to the subject of births; and, on this head, it may be worth while to mention, that the 56th art. of the code provides that every infant shall be presented for registration to the civil authorities of the place, within the third day after its birth; and, in default of this, that every person who has assisted at the accouchement shall be subject to imprisonment of from six days to six months, and also to a fine of from 15 to 300 francs.

Doubtless, all our readers are well aware of the gravely disputed questions so long agitated before the Sorbonne, whether a child should be baptized before its delivery, in cases of apprehended death, and at what period of fœtal life the young being is to be deemed worthy of this consecration, in the event of its premature expulsion. The following amusing extract is from the treatise, "De Hominibus dubiis, sive de Babtismo Abortivorum," of Paul Zacchias, one of the most distinguished casuists of the 16th century:—"Sub peccati mortalis reatu abortivos omnes, quantumvis minimos, etsi phaseolo vel grano hordeaceo non majores, debent baptisari, quantumcunque breve fuerit tempus a conceptione dilapsum: quamvis etiam vitæ signum per motum non prebeant; dum modo corrupti, vel detriti, vel manifeste mortui non dignoscantur."

### On the Agents which may affect the Public Health—Epidemic Diseases.

The third section of medical jurisprudence to which M. Trebuchet leads his readers, comprises what he has designated by the name of "Medecine Legale Administrative," or what is frequently called "hygiene publique." Its aim and object are to watch over and regulate every thing which can influence the salubrity of a place; to observe the varieties of climate, and the influence of these on health; to counteract the effects of all noxious agents, whether on a large or on a small scale; to examine the quality of the various articles of food and drink; to determine the most proper regime for the army, navy, &c.; to suggest the most approved sanitary laws, and to superintend their execution; to report on the state of lunatic asylums, of lazarettos, prisons, cemeteries, &c., and, in short, to examine and ascertain the influence and operation of whatever may affect the general health of communities, or large masses of individuals.

From this imperfect enumeration of the objects of public hygiene, it must appear how extensive and how important this division of legal medicine must be. Our limits permit only a very short allusion to one or two of its themes; and of these we shall select that which has reference to the invasions of epidemic diseases. An example or two will be more instructive than general details.

In 1829, several disorders, having an epidemic character, and occurring without any very obvious exciting cause, were noticed in numerous districts of Paris. As the soldiers quartered in the barracks, in the Rue Mouffetard, suffered most severely, the attention of the medical police was directed, in an especial manner, to the examination of these. All the rooms and wards were found to be in the most admirable order and cleanliness; the food of the soldiers was carefully inspected, and no fault could possibly be found with it; the kitchen utensils, the drains, and, indeed, whatever was considered to have probably any direct or indirect influence on the health of the troops, were submitted to a minute enquiry, and all were declared to be free from any nuisance. At one time, it was supposed that the waters from the fountain at Arcueil were at fault; but then it was remembered that, had this been the case, the surrounding neighbourhood must have suffered as much as the soldiers in the barracks; and the water, when analysed, yielded only its well-known ordinary ingredients. "Il faut, donc (says the reporter), se rejeter sur les causes generales, et notamment sur les variations qu'a subi constamment la temperature depuis quelque tems; sur les transitions rapides du chaud au froid, et du sec à l'humide; sur la fraicheur des nuits qui ont souvent succédé à des journées tres chaudes; enfin sur l'usage de fruits qui n'ont point atteint leur maturité, et dont la vente devrait etre intredite."

The malady in question had many of the characters of cholera morbus; it yielded, however, readily to mild diluents and small doses of opiates. It deserves notice, that the inhabitants in the neighbourhood of the barracks suffered from the same symptoms, but not so severely as the soldiers.

Another report, not less interesting, was made in 1828 on the barracks of Ave Maria, in consequence of a number of the soldiers quartered there having become suddenly affected with tumors and ulcers of the feet, and general feverishness and indisposition, which symptoms were imputed at the time to the unsound quality of the bread used. The affection was a singular one; the soles of the feet became red, hot, painful, and swollen, and exhibited, in some instances, "des callosités tres fortes." In a few cases, the palms of the hands were similarly affected; the skin usually exfoliated after the subsidence of the inflammatory symptoms; the patients were obliged to keep the horizontal position, as any attempt to rest on the feet aggravated their distress. The gastro-enteric system was almost always more or less disturbed. This rather anomalous malady puzzled the medical attendants not a little; but of this they were convinced, that "il ne devoit etre attribué ni à des causes locales, ni à la qualité du pain de munition; il faut plutot en rapporter la cause à l'influence de la constitution atmospherique, dont la nature reste inapercue, et qui se ne devoile que par ses effets." The disease was first noticed about the month of June in the preceding year (1827); it speedily made its appearance in numerous parts of the metropolis, and often attacked every member of a family, without, however, exhibiting very distinct contagious properties. The lower orders of society suffered much more than those in comfortable circumstances, and it seemed to affect chiefly "les artisans et les personnes qui, par etat, etaient exposées à des courses fatiguantes, ou obligées de se livrer à des ouvrages manuels, capables d'exercer une forte pression sur le systeme dermoide."

Its duration was very various, in some cases extending to three and four months. All attempts to dissipate it quickly were wholly inefficacious; and even the mere remedial treatment seemed to be but of little avail. By some physicians, this epidemic was supposed to be allied to the pellagra of Italy; the season of the year at which it broke out, the redness of the parts affected during the first stage, the tuberculous tumors which afterwards made their appearance, the desiccation and desquamation of the cuticle, the prevalence of the disease among the lower orders especially, and its obstinate resistance against every remedial measure—all these considerations might well warrant, at least to a certain degree, the nosological speculation alluded to.

---

### REMOVAL OF PUTREFYING CORPSES WITHOUT DANGER—USE OF THE CHLORURET OF LIME.

In 1830, forty-three bodies of persons killed in the revolution of July, had been incautiously thrown into the vaults of the church St. Eustache. After a few weeks, not only the church, but many of the adjoining tenements, were infected with a most horrible stench. The prefect of the police forthwith appointed a commission, to superintend the exhumation of these bodies: the work was one, not only of considerable difficulty, but of danger at the same time, in consequence of the extreme narrowness of the vaults, and their very imperfect communication with the external air. The members of the commission very wisely resolved to avail themselves of the most potent disinfecting means, and, for this purpose, they employed large quantities of chloruret of lime, both in its dry state and dissolved in water. A huge bucket of the solution was placed on each side of the opening by which the workmen were to descend into the vaults, and others in the body and at the doors of the church. At every stage of their progress, the chloruret was freely used, and so effectual was the precaution, that the stench was scarcely perceptible. Two of the workmen from "La Morgue," and who, therefore, were accustomed to such offensive occupations, being provided with "bridages," descended into the vaults with lighted lamps; the floor and sides of the vaults they first washed with the chloruret, and then MM. Labarraque and Parent de Chatelet followed them, for the purpose of ascertaining the condition of the bodies, and of giving assistance to the workmen, in case they were affected with the putrid effluvia. Much time was spent in clearing away a quantity of earth and rubbish, which had been thrown together in different parts of the vault, and which had partially covered one or two of the bodies; the others had been sprinkled with a thin layer of quick-lime, which had somewhat retarded their disorganization. Several large coarse blankets, well steeped in the chloruret, were stretched out on the floor, and the bodies were then dragged upon them; no sooner was this done, than they were enveloped, and the two open ends secured with strong cords, which firmly bound all fast together: they were then carried to the openings which led to the body of the church, and there again well wetted with the chloruret. During the whole of these most offensive operations, the men repeatedly washed their hands and sprinkled their dresses with the chloruret, while others dashed some against the walls and floor of the vaults. The forty-three bodies were all removed, and conveyed away, without the slightest unpleasant circumstance to any one engaged in the undertaking, which lasted for rather more than three hours. Every thing being completed, "le convois s'est mis en marche à deux heures de la nuit, avec le recueillement respectueux dont l'ame attristé fait une loi, au Cimetiere Montmartre.

---

### Medico-Legal Consultation on a Case of Suspected Infanticide.

MM. Orfila, Ollivier, and Boys de Loury were desired by the "Juge d'Instruction," to examine and report upon a communication which Dr. —— had drawn up, relative to a case of suspected infanticide. The questions proposed for their solution were—Was the infant at the full time when born? Was it born capable of living? Was it born alive? and, lastly, If born alive, had it perished from accident, from wilful violence, or neglect? The body of the infant had been found in a "fosse d'aisance," where it had lain for 21 days. In reference to the first question proposed, the commissioners state—"that the data furnished by the report of Dr. —— are very imperfect and unsatisfactory: it is mentioned "that there were several patches of hair, an inch and upwards in length, on the scalp—that the nails of the fingers and toes were well formed —that the navel-string was of the length and thickness usual in infants at or near the full time—that the placenta had not been discovered—that the extremities and other parts of the body, which had not been mutilated, appeared to be normal in size and formation, although somewhat enlarged, from the effects of putrefaction, and the consequent cadaveric imbibition." The defects of this report are, that neither the length nor the weight of the body are stated; that the mutilations of the body must have prevented Dr. —— "de reconnaitre le point d'insertion du cordon ombilical" (?); that the length and size of the navel-string are too variable to warrant a very decided opinion that the child was nine rather than only seven or eight months; that Dr. —— has omitted to ascertain, whether the epiphysary cartilage of the os femoris exhibited a nucleus of ossification. The absence or insufficiency of these and other particulars prevents us (the commissioners) from giving a positive answer to the first question, and can only justify the allegation, that it is probable that the infant had reached the seventh month at least, at the period of birth.

2. Was the infant, when born, capable of living (viable)?

To this second question, Dr. M. has replied—"that it is impossible to decide this point, in consequence of the advanced state of putrefaction, and the horrible mutilation of the body; and also because there might possibly have existed some defect, or other error of organization incompatible with life, or some disease essentially and speedily mortal, although neither of these states could be ascertained, for the reasons now mentioned." But certainly there are no data in the report to warrant either of these suppositions. On the contrary, we are bound to believe, in spite of the mangled and putrid state of the head, that the child was not anencephalic; and, indeed, it is expressly stated, that the cerebral substance was converted into a white, curdy, almost fluid pap, and that all the cranial bones could be perfectly well recognized; the description of the heart and lungs shews too, that these organs were quite normal in their development; and no distinct malformation was discovered in any part of the body. We cannot, therefore, admit, with Dr. ——, that the putrefaction and mutilation of the body can be pleaded for the conjecture, "that some congenital lesion or disease, incompatible with life, might have existed, although not discoverable on the examination of the body." We are, therefore, of opinion, that no satisfactory reasons exist for believing that the "enfant ne fut pas viable;" and we may add that, if the report of the appearances found on dissection had been more minute, we might probably have been able to decide more definitively on the question of the viability.

3. Was the child born alive?

This question, which Dr. —— has left unanswered, may be, in some degree at least, elucidated by the following paragraph of the report. "The cavity of the thorax contained no fluid; its viscera exhibited no traces of any lesion; the lungs were ash-coloured on the outer surface, exceedingly small, and lay shrivelled up on the vertebral column; the left lobes, concealed behind the heart, were altogether like the lungs of a still-born infant; nevertheless, they crepitated somewhat on pressure, and, when divided with the scalpel, they presented a surface of a deep port-wine colour; they floated in water, but no inference could be drawn from this last-mentioned circumstance, as the liver, and even the heart, were almost equally buoyant."

Doubtful, indeed, as the question must be, we are of opinion that the shrivelled, collapsed state of the lungs, and the dark sanguineous colour of their cut surfaces, should lead us to the belief, that the act of respiration had never taken place. The absence of meconium in the rectum, and also in a considerable extent of the colon, does not invalidate this opinion, for the discharge of this intestinal matter may have been induced in several ways; thus, in a hip-presentation, it is not of unfrequent occurrence, that the meconium is voided during the act of labour, especially when this is tedious, and the child is detained long in the pelvis. Perhaps even the compression of the body of the child, during the repeated attempts to drag it out from the pipe of the "fossé d'aisance," may have contributed to the same effect. It may be added, that the total absence of the usual green colour in the contents of the upper portion of the large, and in the small intestines, is a circumstance which may induce us to suspect that the infant was not at the full term when born, and which may, therefore, be mentioned as confirming the supposition, that it was not born alive.

4. If the child was born alive, what was the cause of death?

The answer to the former question has anticipated our reply to this one. The mere fact of the child's body exhibiting numerous injuries and mutilations cannot be fairly adduced, as proving that its death had been occasioned by violence; for it is not impossible that these injuries and mutilations were inflicted during the attempts to extract the body from the pipe of the privy. Nevertheless, it would have been most desirable that the description of these injuries had been much more exact and minute than has been given in the report, as it is possible that it might thus have been determined whether they had occurred before or after death.—*Archives Generales.*

---

### Poisoning with Belladonna.

During the choleraphobia which prevailed so generally over France in 1832, various nostrums were recommended and used as preservatives against the mysterious foe. Among these was an infusion of the leaves of the menyanthes trifoliata, or common buckbean of the fields. One morning, in the month of July, Dr. Claubry was summoned to the relief of an elderly man, who, it was supposed, had been seized with an apoplectic fit. On entering the apartment where the patient lay, he was much struck with the peculiar physiognomy of the younger daughter of the family; her look was dull, vague, and unsteady, almost like that of a person labouring under amaurotic blindness, and her features were affected with a curious unmeaning smile, which was evidently involuntary: the voice sounded as if it were masked, the gait was unsteady, and the girl could not stand erect without laying hold of some support.

Dr. C. made no enquiries at the time, but proceeded to examine the state of the father, who was lying in bed:—His face was of a purple hue; the conjunctivæ injected with a bluish-coloured blood; the pupils dilated and immovable; the lips and tongue parched; the man complained of intolerable thirst, and of a distressing feeling of constriction in the throat; his speech was embarrassed, and scarcely intelligible; the skin was warm, the pulse full, and rather slow. The two daughters reported that they had called their father that morning at 8 o'clock, but that they found him sleeping unusually heavily; that, when they awoke him, he seemed to be confused and scarcely conscious; that his voice was thick and interrupted, and that, when he attempted to rise, he became giddy, and complained of every thing going round with him. The correctness of these details was confirmed by Dr. C's. own examination, and he was now struck with the resemblance in the expression of the younger daughter, to that of the father, although in a much less degree. He immediately suspected that they were both suffering from the effects of intoxication; and his suspicions became stronger, when he took notice of the elder daughter, who was similarly affected with a general stupidity and confusion. " La station difficile; demarche incertaine, air d'etonnement, d'hebetude, œil inanimé, parole entrecoupée, secheresse de la langue, de la bouche, des levres," &c.; and if we add to these, an unusual loquacity, a sort of foolish unmeaning smile, my readers will not be surprised, says Dr. C. that I at once exclaimed " Dieu me pardonne, vous avez tous les trois une pointe de vin!" But the doctor was soon satisfied of his error; and on making further enquiries, he found that they had all drunk several cups of the supposed buckbean tea, on the preceding evening, before going to bed. The remainder of the dried herb was now examined, and Dr. B. at once recognised that it was certainly not the menyanthes; the packet had been bought at a " petite boutique assez mal tenue d'herboristerie." Without, however, losing time, to ascertain what plant it was, Dr. B. ordered all three to drink very copiously of lemonade, to have mustard poultices applied to the extremities, and to be kept awake, and if possible, moving about the room. Fortunately these means were sufficient, and in the evening, the symptoms of oppression had greatly decreased in every one. It may be worthy of notice, that no cutaneous redness was observable in any of these patients, although all of them complained of considerable tingling and sense of pricking over the surface of the body.

In two cases lately reported by M. Laurand, a distinct " eruption scarlatiniforme" was perceptible, and more especially on the body and thighs. The following is a brief detail of the particulars.

Some pills containing a minute quantity of the extract of belladonna had been ordered for two young children, who were suffering from hooping-cough. The chemist had mistaken, we are told, the word "grains" for " gros" (!) and a poisonous dose of this most active drug had therefore been inadvertently administered. For several hours the children were exceedingly drowsy, and slept very heavily. On awaking, they were observed to squint, and to talk confusedly of all sorts of foolish things, calling out that they saw rats and mice running about the floor, butterflies on the walls, beautiful birds flying about, candles burning, stars sparkling, and so forth.

The loquacity of the children, both of whom were naturally of a dull taciturn disposition, was very remarkable. This " espece de delire jovial" continued for three or four hours, and was again succeeded by a tendency to drowsiness. By the use of acidulated drinks and the application of sinapisms to the stomach and extremities, all the unpleasant symptoms subsided, and on the following day, the children had quite recovered from the effects of the poisoning. The hooping-cough, however, " reparut de la meme intensité." From the preceding observations, we may infer, that the action of belladonna, in doses not so poisonous as to prove fatal, is to produce, first a stupefying effect, and then an " exaltation de toutes les facultés."—*Journal Hebdomadaire.*

---

### Laceration of the Diaphragm—Medico-Legal Enquiry.

In last September No. of the Archives Generales de Medecine, an interesting case of this most alarming accident is recorded, and some valuable remarks are appended to the report by the narrator, with the view of shewing that although almost necessarily fatal, it may not be immediately so, and that the patient may survive for several hours. Three men returning half drunk from a fair, met a labourer on the road, whom they began to insult, but whom they soon found was more than a match for them all. After they had received a good drubbing, they made the best of their way to Dr. Davat's house, a distance of rather more than half a league from the scene of the scuffle; they took a full hour to reach it. Two of the men were found to have received only some trifling bruises; but the third one, more advanced in years, and who had sat down when he entered the room, resting his head on his hands, and his elbows on his knees, seemed to have been much more severely injured; his features indicated inward distress, but not certainly of a very alarming character. After he had rested a few minutes he rose, walked slowly, but without tottering, to the door. Hitherto he had had no vomiting, nor had he uttered a syllable or even a moan, but had remained all the time in the attitude we have just mentioned. When they left Dr. D.'s house, they had an hour's walk before they could reach their own homes; at this time it was about six o'clock P.M. Several people joined them on the road; but the elder of the three continued in the same taciturn absorbed state; he would not speak, and answered questions only by "yes" and "no." Still he made no complaint of any severe pain or suffering. Their progress must have been very slow, for we find that at nine o'clock they had gone only half their journey; and then J. V. (the eldest) found himself unable to proceed any farther; exhausted, he lay down in an outhouse, and soon becoming insensible, he remained there till the following morning, when he was carried home. Dr. D. found him lying on his back, in a completely comatose state. No external traces of any severe injury could be discovered any where; "le ventre, souple, n'avait rien de particulier." It was therefore conjectured that the encephalon had sustained some injury; probably that some vessel had given way, and that effusion had taken place to a limited extent. A vein in the arm was opened, but no blood could be obtained—he died at 1 P.M. The dissection was performed by the authority of the civil magistrate, in consequence of the report that the patient had died from the bruises he had sustained in the scuffle. On dividing the scalp, a quantity of black blood was found effused into the cellular tissue between the occipito-frontalis and the bone, and also in the interstices between the muscles on the back of the neck. Over the middle of the left parietal bone, there was a limited extravasation, resting on the bone itself; the sub-pericranial cellular tissue was somewhat detached: on clearing the blood away, a double fracture was found; one running longitudinally, the other transversely, and both meeting at the "bosse" of the parietal bone; from this point a small fissure communicated inwardly with the cavity of the cranium, and it had been along this channel that the blood extravasated under the pericranium had made its escape. On elevating the bone, an effusion to the amount of nearly two ounces was discovered on the surface of the dura mater, which was detached from the bone, for at least five inches in extent. When the thorax and abdomen were opened, a very remarkable lesion was found. A lacerated opening through the centre of the diaphragm had given passage to the large cul-de-sac of the stomach into the cavity of the chest; the rent was two inches and a half long, and had certainly been of very recent occurrence, as the edges were still tinged with blood; the extruded portion of the stomach also was ruptured, and the contents of the viscus, consisting of half digested meat, vegetables, bread and wine, and some clots of blood, were found effused into the thorax.

*Remarks.*—It was necessary for the medical men who had attended this patient, and had examined the corpse, to report an official account of their enquiries, and to declare their opinions as to the probable cause of death. With regard to the injury of the head, the following very obscure and puzzling decision is given. "Dans quelque position que se trouve le corps, une pareille chute sur le parietal est impossible. La violence qui a fracturé le crane n'a donc pu etre que l'effet secondaire d'une premiere cause qui a mu cette violence en sens inverse de l'attraction !"

The other important injury, that of the diaphragm, may give rise to much greater discrepancy of opinion, as to the cause which produced it, and the exact time at which it happened. It has been a very general, and we must add, a very natural belief that such an injury as this, and especially when it is complicated with another, perhaps still more mortal accident, we mean that of rupture of the stomach and the consequent extravasation of its contents, has always been very speedily fatal. Let us analyse the particulars (as far as they can be gathered from the perhaps garbled reports of the surviving companions) of the present case, before we decide.

It is alleged that the man had been severely beaten; that he was stunned in consequence of the blows received, but that he was not at any time quite insensible or comatose; that on recovery from the stunning, he walked the distance of half a league, slowly indeed, but sometimes without any support, or assistance, and that he scarcely spoke a word, the only answer which he ever gave to enquiries, being by a simple "yes," or "no;" that when he entered the room of Dr. D. he sat down, and remained in a bent or folded position all the time he was there; that he made no urgent complaint, but seemed stupid from the effects of intoxication; that his breathing did not appear to be particularly distressed; that he arose from the chair by his own efforts, and that his walking as far as the mill, where he lay down, was "lente, grave, mais non mal assurée." During all this time, he had never been completely insensible, nor had he ever vomited, although he had made several attempts to do so. Now all this history is quite intelligible, had there been no other accident, except that of the cranium; for we might suppose that the effusion of blood had been slow and gradual, and the natural effect of this must have been to have caused a lethargic stupor, which blunted the pain and distress, which otherwise must have been experienced; but we can scarcely admit that the lesion of the diaphragm could have taken place at the same time, and that so serious an accident could have been compatible with the prolongation of life, for so many hours, as occurred in the present case. Some light may be thrown on this subject, by examining the records of similar instances. Baron Percy is one of the best authorities on this subject, and some important observations have been published by him in the art. Diaphragme, in the Dict. des Sc. Med. and also in M. Cavalier's Inaugural Dissertation. We shall extract a few of the cases detailed in these memoirs.

A carpenter fell from the dome of the Invalids upon some scaffolding. He gradually recovered from the effects of this accident, but ever afterwards was troubled with a frequent cough, dyspnœa, and a pungent pain on the left side of the chest. Six months afterwards, he was so unfortunate as to fall from a height of twenty feet on the ground, and he broke seven of his ribs. He lived only for four days.

On dissection, there was found an opening, two inches and a half in extent through the aponeurotic centre of the diaphragm; the edges of this opening were cicatrized; a portion of the stomach and also of the colon had become protruded into the cavity of the thorax; the heart was pushed to the right side, and the left lung was much shrivelled in size, and pushed up to the top of the chest.

A young woman was suddenly affrighted when the pains of labour were on her. She uttered a scream, spoke a few stifled words, and expired. The diaphragm was found ruptured in its left side, and a large portion of the stomach, epiploon, and colon had escaped through the opening into the cavity of the thorax.

In the other three cases related by the Baron, death took place almost instantaneously; in all of them, the accident seemed to have been occasioned by violent efforts of vomiting.

A strong athletic man had gone down into a vault, for the purpose of assisting to lower a coffin into its place; he was standing underneath it, while other two men were easing it down from above. Afraid that it would fall upon him, he was trying to stop its too rapid descent, but at that moment he dropped down insensible, and after a few groans he expired. A considerable laceration through the substance of the diaphragm was found on dissection.

It will be remarked, that in all these cases, except in the first, the death of the patient was almost immediate, and in all, the symptoms of some mortal injury were strongly enough marked during the short interval of life; the pain, the inward agony, the ghastly faintness, and the dreadful oppression of breathing were invariably more or less present. How different then from the history of the case, now under consideration! The conclusion, therefore, which we feel ourselves bound to deduce, is ' que J. V. pouvait, peutetre tout au plus, avoir le crane fracturé lorsqu'il s'est presenté chez M. Davat, mais qu'il ne portait pas l'ensemble des blessures qui ont été decrites."

[When we had drawn out all the details of the present case, we chanced to light upon a paper in a recent number of the Journal Hebdomadaire, in which reference is made to one somewhat analogous, in which the patient lived for six days after the accident. It is recorded in the Journal General de Medecine for 1819. A healthy and vigorous man, 59 years of age, was attempting to mount to his seat on the top of a coach: he had one of his feet on the front wheel, the other resting on the ground; and just in the act of lifting himself up, he pulled the coach, (which must have been very light) over and fell back, underneath it. He was taken to the Hôtel Dieu on the third day after the accident; the right thigh was found to be fractured; but no alarming constitutional symptom was present; the pulse was natural, the respiration not oppressed; and the only annoyance was a teasing cough, accompanied with a copious expectoration. He unexpectedly sunk on the sixth day, " apres une agonie courte et peu douloureuse." The dissection quite surprised all the medical men; for no sooner was the sternum lifted up, than a portion of the intestines escaped from the thorax; the left lung was squeezed up to the top of the thorax; the diaphragm had been detached from its adhesion to the sternum and ribs for a considerable extent, and through this large gap the bowels had been protruded; there was also another smaller rupture of the diaphragm, beginning at its point of attachment to the last true rib of the left side, and extending to the corresponding crus of the muscle.—REV.]

In what manner, and from what mode of injury, the accident was produced, it is quite impossible to determine. No satisfactory information could be derived from the testimony of the man's companions, and we have only the negative evidence of the absence of all external bruises of the skin, and of any mud, or impression of other substances on the clothes of the chest and belly. " La rupture,' says M. D. " du diaphragme, et de l'estomac ne peut elle meme s'expliquer que par l'action d'une violence exterieure qui s'est exercée en repoussant la masse intestinale de bas en haut. Le corps qui a agi a dû luimeme etre recouvert de substances souples; il a dû agir tres promptement avec une violence extraordinaire, et d'un seul coup, car les parois abdominales ne presentent aucune lesion. Quel corps reunit mieux ces conditions que le genou?"

With the view of throwing as much light as possible on this obscure subject, M. Davat performed a good many experiments on dead bodies for the purpose of ascertaining whether the stomach when distended with food, the œsophagus being tied, may be ruptured after death, by a heavy blow on the abdomen. He found that a single blow was very rarely sufficient; for only once, in six subjects, did it succeed, and in this case the stomach was literally filled to cramming: but a repetition of blows was always effectual, provided the viscus was not empty; sometimes it was ruptured in two places at the same time. In none of these experiments was the diaphragm ever found injured; probably from the circumstance of the lungs being uninflated, and there being therefore room above for the diaphragm to be pushed upwards. During life, on the other hand, this moveable curtain is pressed upon, on both its sides, by the distended thoracic and abdominal viscera; and if we wish to perform experiments on the dead subject, which can have any analogy with accidents occurring to the living, it is necessary that the circumstances of the latter state be imitated as nearly as it is possible. With this view M. D. distended the intestines and stomach with air, and secured the œsophagus and colon with ligatures; he next inflated the lungs and tied the trachea; and having given several severe blows on the epigastrium, he found, on opening the body, that not only the stomach, but the diaphragm also was ruptured, without however any protrusion of the abdominal viscera through the aperture. Not satisfied with this result, he made some trials on living animals, and the evidences of these agreed so far with the results which he had already obtained, as to convince him that the lesion produced depended in a great measure on the condition of the respiratory organs at the moment of the infliction of the blow. If expiration has taken place, the stomach only was usually ruptured; but if the lungs were inflated, the diaphragm also was sometimes ruptured, and the stomach found protruded through the opening into the thoracic cavity. Such experiments as these are infinitely more satisfactory (although we cannot but condemn such gratuitous cruelty) than any performed on dead bodies; but we must confess that we are somewhat puzzled to account for the double rupture in one of the trials on the dead body. If quite correctly stated, it must have been caused by the violence of the blow, and by this alone; but until we have further data, we shall continue to hold the opinion, that rupture of the stomach is the effect of a vital, rather than of a mere mechanical action. It is much more reasonable to attribute such a lesion, when it is found after a sudden death, to a spontaneous laceration from a tetanic spasm (a cause which we know is quite sufficient to induce rupture of a muscle) than to the mere agency of a direct blow however violent. There was recently a case in the Cochin Hospital, of rupture of the diaphragm, in consequence of a blow with the fist on the left side of the thorax. It is not surely probable that the mere mechanical force of the blow was the immediate cause of the rupture. In concluding the report of the preceding most interesting case, it may be worthy of mention, that the court, before which it was examined and tried, " faisait porter tout le poids de l'accusation sur l'homme qui a battu J. V. à la foire ; mais qu'admettant des circonstances attenuantes la peine a eté reduite à sept ans de travaux forcés."—*Archives Generales, Sept.* 1834.

---

### Appeal of the Principal Surgeons in Paris against the Violation of Hospital Appointments by Concours.

The following petition addressed lately by the members of the central bureau of hospitals, to the Minister of the Interior respecting a recent official appointment, is worthy of being recorded, as a proof of the resolutely honorable feelings which pervade the medical profession in France.

M. Le Ministre.—The office of Surgeon to the Maison Royale of Health in the Faubourg St. Denis, has become vacant, by the resignation of M. Jules Cloquet. One of the physicians of that establishment has applied to the Council-General of Hospitals, for permission to change his title of Physician to that of Surgeon, and to be appointed to the vacant office.

Such a change being unprecedented, and contrary to the fundamental provisions of the law, the Council-General has resolved to refer the question to the Minister of the Interior. The undersigned surgeons of hospitals, and of the Central Bureau, deem it their duty, as being interested in the welfare of the public, and in the maintenance of individual rights, to address to you the following observations.

When the office of surgeon to any of the hospitals becomes vacant, there are only two legitimate modes of filling up the vacancy; either by directly naming a surgeon, or by imposing the duties of the vacant office on a surgeon of another establishment.

The 24th* Article of the Code, regulates the nominations in the one case, and the 5th† Article determines the limits, within which, in the other case, the changes can properly be made.

The appeal of the Council-General is manifestly in opposition to the enactments of these two articles, and this violation, if permitted, will introduce confusion into the medical arrangements of the hospitals.

It will encourage others to use the same subterfuge, in attempting to obtain the office of surgeon, without undergoing the ordeal of the Concours, an ordeal which has been always recognized, as affording the only sufficient guarantee for the qualifications of candidates.

It will have the effect of withdrawing surgical appointments from the surveillance of the Minister of the Interior, and vesting them entirely in the Council-General.

It will deprive those surgeons who are at present surgeons of other hospitals, of their right of change, a right which they may have acquired by the length and value of their services.

It will also unfairly stop the career of the surgeons of the Central Bureau, who have acquired, by their examinations at the Concours, and by their services in the hospitals, the right of being elected to vacant offices.

Relying upon the justice of their reclamation, the undersigned surgeons of hospitals and of the Central Bureau, petition you, M. le Ministre, to order the Council-General to act strictly according to the existing laws.

Monod, Robert, Michon, Guersent, Vidal, Danyau, Surgeons of the Central Bureau.

Appended to the foregoing petition are a number of certificates or declarations from the leading surgeons in Paris. We shall select a few, and give them in their original.

"La violation de la loi du concours, loi si sagement ètablie par le conseil-general des hôpitaux, serait un coup funeste porté a la medecine et a la chirurgie des hôpitaux, et par conséquent aux malades qui viennent s'y faire traiter; elle indiquerait à tous ceux qui voudraient y être admis comme médecins ou comme chirurgiens, qu'ils ont moins besoin de savoir que d'intrigues.

DUPUYTREN.

"La reclamation de MM. les chirurgiens du Bureau central me semble tout á fait fondée sur le respect qui est dû aux droits qu'ils ont acquis. La mesure projetée, et contre laquelle ils s'élèvent avec raison, me parait contraire à l'équité, et peut-être tout à la fois nuisible aux intérêts de l'enseignement et à ceux des malades.

PAUL DUBOIS,
*Professeur à la Faculté de médecine.*

"La réclamation adressée â M. le ministre de l'intérieur par MM. les chirurgiens du Bureau central est fondée sur la raison et sur le texte des reglemens actuellement en vigueur. Il serait désespérant pour l'avenir que le conseil-général des hôpitaux transgressât une loi que lui-même il s'est imposée. Je prends donc la respectueuse liberté de recommander à M. le ministre la demande qui lui est adressée.

Paris, 6 decembre 1834. ROUX.

"La présente réclamation est fondée sur la raison et l'équitê, ainsi que sur les réglemens d'apres lesquels, jusqu'à ce jour, on a fait les nominations dans

---

* The physicians and surgeons of hospitals and of hospices (institutions for the aged, destitute and incurable) shall be appointed by the Minister of the Interior, on the recommendation (avis) of the Prefect of the Seine, from a list of three candidates, selected by the Council-General.

† The physicians, surgeons, and apothecaries of hospitals and hospices, may upon application, and with the permission of the Council-General, be tranferred "en la meme qualité," from one establishment to another.

les hôpitaux civils de Paris. Si elle n'était pas écoutée favorablement par l'autorité supérieure, ce serait un précédent déplorable. On ouvrirait ainsi une porte á l'intrigue, à l'ignorance; on paralyserait le zéle et l'émulation des jeunes chirurgiens, et l'on verrait bientôt la direction de nos hôpitaux confiée à la médiocrité, et tout enseignement détruit dans ces asiles de consolation pour les malades et d'instruction pratique pour les élèves.

BRESCHET,

*Chirurgien ordinaire de l'Hôtel Dieu.*"

"Je partage absolument l'opinion exprimée par mes honorables collègues, et ne crains pas d'ajouter que ce serâit un antécédent des plus dangereux et des plus injustes à la fois que de permettre la mutation contre laquelle ils s'élèvent.

VELPEAU."

"La demande adressée à M. le Ministre me semble de nature à devoir être approuvée par tous les amis de la justice. Je crois, en mon particulier, que la mesure contre laquelle elle est dirigée serait attentatoire, de la manière la plus manifeste, aux droits acquis de MM. les chirurgiens du Bureau central.

BLANDIN,

*Chirurgien de l'hôpital Beaujon.*"

"Je partage entièrement l'opinion de mes collègues sur la demande de nos jeunes confrères du Bureau central. Ils réclament un droit acquis par le concours et par le réglement des hôpitaux. Qu'on y prenne garde; on établirait un précédent funeste qui livrerait les pauvres malades à l'ignorance et à la médiocrité.

LISFRANC,
*Chirurgien en chef de la Pitié.*"

"La réclamation de MM. les chirurgiens du Bureau central me parait parfaitement fondée, et je me joins avec empressement à mes confrères pour prier M. le ministre de l'intérieur d'y faire justice.

Baron RICHERAND."

---

SOUVENIRS DU CHOLERA.

Such is the heading of one of the themes, in a series of satirical poems on medical subjects, entitled "Nemesis Medicale, ou Recueil de Satires, par un Phocéen," which has been recently published in Paris. Some of these squibs in rhyme are sufficiently amusing, and evince considerable talent for droll and piquant detail. The Ecole, Academie, le doyen de la Faculté, Conseil Royal de l'Université, the hospitals, professors, &c. all have the whip applied to them in their turns, now with a mere good-natured fillip, and now with the biting lash of severity. The author promises, in the outset, that he will keep "sa verve" within the bounds of decency and fairness.

"Quelque soit le champ clos ou notre ardeur se joue,
Jamais nous ne prendrons nos rimes dans la boue."

As the exordium to the "Souvenirs du Cholera" evinces "noblesse de pensée, pureté d'expression, et richesse de la rime," we subjoin it, for the benefit of the more prosaic "medecins" on this foggy side of the Channel.

"A quel temps de douleur vais-je, helas, m'inspirer?

Du dueil universel je me sens penetrer;

Et sensible à ma voix, emue à mes alarmes,

Nemesis, elle-meme a repandu des larmes.

Comment ne pas pleurer dans nos murs consternés,

Sur vingt mille habitans en vingt jours moissonnés;

Sur ces tristes debris d'immenses hecatombes,

Sur ces monceaux de morts dont regorgent nos tombes?

Medecins, dans mon cœur saisi d'un saint respect,

Mon sang vivifié tressaille à votre aspect;

Vous pour qui le public s'est fait une habitude

Du dedain, de l'injure, et de l'ingratitude.

Nuit et jour au chevet d'un malade expirant,

Humant du cholera le souffle devorant,

On vous vit defier sa menacante approche,

Bayards incuirassés, sans peur, et sans reproche;

Par votre devouement et votre autorité,

Vous avez rassuré le peuple epouvanté.

Prompts à jeter vos corps en gage de bataille,

Vous n'aviez pas alors l'espoir d'une medaille."

We hope that "le sang vivifié" of our readers may "tressaillir" when they peruse these laudatory strains.

In his endeavour to clothe the technical description of this modern plague with the graces of verse, our author has displayed "un tour de force," which is sometimes really very happy; for example—

"Vous cherchez vainement dans ce poignet perclus,
Une artere qui fuit, un pouls qui ne bat plus.
Tout en lui, tout est froid; chez ce mort qui respire
La chaleur bienfaisante a perdu son empire;
Et quand il fait revivre un corps ainsi formé,
Dieu d'un souffle nouveau doit l'avoir animé."

---

### ON THE RESPONSIBILITY OF MEDICAL MEN IN FRANCE.

At Rome, the Lex Aquilia decided that medical men were responsible for errors committed in the pursuit of their professional duties, whether these errors proceeded from ignorance or from inattention. No one had any right to complain of this enactment in those early times, seeing that there was an authoritative codex, containing a certain number of prescriptions, from which physicians and surgeons were ordered not to deviate. This prohibitory code was, as a matter of course, repeatedly neglected, and it gradually fell into desuetude. The earliest records of medicine in France prove that a lex Aquilia was recognized in that country. We have the account of the trial of a surgeon, in April, 1427, for having administered "un remede violent, qui pouvait le tuer ou le guerir en peu d'heures." In the records of the French Parliament for 1696, we find, however, "que les chirurgiens ne sont pas garans et responsables de leurs remedes, tant qu'il n'y a que de l'ignorance, ou de l'imperitie de leur part, quia ægrotus debet sibi imputare cur talem elegerit." In 1716, a surgeon was amerced in 15000 liv., to be paid to a young man whose arm required amputation, in consequence of a maltreated fracture, and he was forbad to practise surgery in future. In 1725, a surgeon of long standing, and of acknowledged skill, was so unfortunate as "estropier un malade en lui faisant une incision." No charge of negligence or incapacity could be laid against him, but "he had operated without the sanction of two other surgeons, who had been called into consultation with him."

The defence rested on the inapplicability of the expression, "si male et imperite," to the accused; and it was fairly urged, that many diseases and accidents terminate unfavourably, in consequence of the unhealthy constitutions of the patients, and of other accessory circumstances, such as the state of the weather, mental emotions, &c. The court nevertheless found the surgeon liable; but, in consideration of his long-tried ability, and of his good intention, "a bien voulu ne pas donner un jugement rigoureux—elle vous enjoint d'appeler à l'avenir un conseil dans les grandes cures, et soit que vous soyez l'ancien, ou le plus jeune, de déferer à l'avis de la majeure partie dans la consultation qui sera faite." The responsibility of medical men is to this day, in France, not founded on any appropriate and definite law, but on certain articles of the code, which are of a very vague comprehensiveness, such as the following:—"Quiconque, par maladresse, imprudence, inattention, negligence, ou inobservation des reglemens, aura commis involontairement un homicide, ou en aura eté involontairement la cause, sera puni de trois mois à deux ans, et d'une amende de 50 francs à 600 fr.;" and "chacun est responsable du dommage qu'il a causé, non seulement par son fait, mais encore par sa negligence, ou par son imprudence." Of late years, not a few trials have been instituted against medical practitioners for imputed mala praxis, and heavy damages have been awarded in several instances. In 1825, Dr. Helie was called to the assistance of Mad. Foucault in labour: the labour was long, and very tedious, and Dr. H. supposing that the child was impacted in the pelvis, cut off both arms, which protruded; the child unfortunately survived the delivery. The father prosecuted the Doctor; the Doctor in vain urged that a "Docteur en Médecine et en Chirurgie" was not responsible for "ses faits de pratique." The Court laid the case before the Royal Academy of Medicine, a committee of which examined all the statements most minutely, and reported "que l'accoucheur avait commis une faute, mais qu'il n'appartenait pas à l'Academie de prononcer s'il devait en etre responsable." When this report was made to the Academy, objections were raised against it on several grounds, and ultimately the following declaration was agreed to;—"la manœuvre inculpée compte pour elle un assez grand nombre d'autorités imposantes, pour qu'elle ne puisse pas etre attribuée à l'erreur." Somehow or other, another commission was appointed, and the judgment which the members pronounced was, "que l'Academie ne trouvait, dans les pieces de la procedure, aucun element suffisamment clair pour repondre aux questions du tribunal." But the learned jury of Domfront were not to be baulked in this way; they did not hesitate to say that the questions had been "eludées plutot que resolues" by the Academy, and forthwith they condemned "le pauvre Docteur" to pay to the child an annuity of 100 francs for the first ten years of its life, and afterwards one of 200 francs during the remainder of its life; all the expences of the action were also saddled upon Dr. H. This case excited the greatest interest among medical men in France, and gave rise to numerous and very acrid disputations. As connected with obstetrical jurisprudence, it may be worthy of notice that, by the article 33, "midwives are forbidden to employ instruments, in cases of difficult labour, without the concurrence of a recognized medical man."

Three years ago, Mad. Durand was treated by "le Sieur Charpentier, officier de santé," for a supposed fracture of the carpal ends of the anti-brachial bones. Under this impression, he applied splints and bandages; the limb began to swell, became inflamed, and ultimately gangrenous: the eschars, indeed, separated, and the sores healed; but the hand was permanently bent upon the forearm, and the member was left utterly useless to the patient. The consequence of this misfortune was, that the patient was reduced to beggary. After a lapse of three years, Charpentier was prosecuted, and amerced to the amount of 4000 fr., "comme coupable de blessures graves, commises par imperitie ou par imprudence." [The error committed by M. C. in this case is of very frequent occurrence; we recommend our readers to consult a paper at page 216 of our last No. where they will find several instances detailed illustrative of this remark.—Rev.]

An appeal was made from this judgment, and Dr. Olivier was appointed to examine the woman's hand, and report his opinion upon the case: he "declara qu'une luxation du radius etait un de ces accidens graves qui necessitent, de la part d'un officier de santé, l'appel d'un medecin comme conseil, et que l'estropiement de la femme Durand provenait moins de cette luxation, que de la constriction par les bandages." [A proof of the strange and anomalous state of the medical profession in France.—To make it truly respectable, all, the officier de santé, as well as the médecin or docteur, must be equally educated, and all equally responsible.—Rev.]

The "affaire de M. Thouret Noroy," who was lately amerced in a heavy fine, for imputed mala praxis, deserves a short notice. M. N. was so unfortunate as to wound the brachial artery during venæsection: an aneurismal varix formed; the patient applied to another surgeon, who, most unhandsomely and most unprofessionally, performed the operation of tying the artery, without acquainting M. N. of it. The operation was unsuccessful; and a gangrenous inflammation having appeared in the fore-arm, amputation of the limb was necessary: the unworthy M. Chouippe was again the operator.

On recovering from these complicated distresses, the patient sued M. Noroy for damages. The tribunal of Evreux awarded 600 francs, and also an annuity of 150 fr. to the prosecutor. M. N. appealed from this decision to the "Cour Royale" at Rouen; but, instead of obtaining what he considered a just redress of his wrongs, the fine was raised from 600 to 1000 francs.

He has now addressed himself to the Royal Academy of Medicine at Paris; but this liberal corps has, with courtier cunning, taken no notice of the appeal.

In consequence of this cruel treatment, a large meeting of the "Association des Medecins de Paris" was recently held, to determine what steps should be taken in favour of M. Noroy. The substantial relief of pecuniary subscription was not neglected; and M. Dubois, the Honorary Dean of the Medical Faculty, commenced the list by a noble donation of 500 francs.—*Jurisprudence de la Medecine.*

---

### FEES OF MEDICAL MEN—A LEGAL CLAIM IN FRANCE.

Although the legality of the claims of a medical practitioner is recognised, there is much uncertainty, and frequently not a little disputation, as to the extent to which this legality may be urged. The art. 2101 and 2272 of the civil code are the only ones which refer to this subject, and they do so only imperfectly: "Le premier, en declarant privileges les frais quelconques de derniere maladie, concurremment avec ceux auxquels ils sont dues, et le second, en prescrivant par un an l'action des medecins, chirurgiens et apothicaires pour leurs visites, operations, et medicamens." It will be observed that according to the former of these articles, the claim is classed along with the other expenses of the "derniere maladie;"—no reference is made to other illnesses, nor does it satisfy the question whether the medical practitioner be entitled to rank before, and in preference to, the other creditors, in the event of a failure. "Les tribunaux," we are told, "ne paroissent point admettre cette interpretation, (granting to medical men any peculiar "priviligium" over the other creditors); ils se fondent sur ce qu'il est question de ce privilege immediatement apres les frais funeraires, et ils en concluent qu'il n'existe que *lorsqu'il y a mort du malade.*"

The conclusion therefore must be that "les creances des medicins ne sont *privilegiées* qu'autant qu'elles s'appliquent à la derniere maladie. Les honoraires qui seraient dus pour des maladies anterieures ne seraient l'objet d'aucun *privilege.*" The term of twelve months is allowed to make the claim; when the period, elapsed since the attendance, has exceeded that time, the claim for any remuneration has often been disputed. We shall now very briefly allude to the amount of charges which the French law awards to a medical practitioner. Our author says, "En reconnaissant aux medecins le droit de reclamer leurs honoraires en justice, la loi veut que ces honoraires soient proportionnés à l'importance du traitement, et surtout à l'etat de fortune du malade, ou de ses heritiers." In a late trial, a Dr. B. made the following claim against one of his patients, Mad. L. "Deux ans, deux mois, douze jours, à trois visites par jour, font 2046 visites, à deux francs, 4812 francs."

In addition to this charge, there were some others, for having applied at different times blisters, cauteries, dressings, &c.; for operations on the toes, for venæsections, for consultations, for sleeping all night in the patient's house, and "enfin pour cent cinquante vacations à l'immersion des bains de la malade;" not to mention many other acts of professional kindness, "afin," says the considerate doctor in his letter of claims, "de ne point leser la part de mon affection." The patient had offered to pay 2400 fr. The Court, after hearing the evidence on both sides, deemed this sum insufficient, and "condamna M. L. a payer 4200 fr. sous la reduction de 750 fr. pour la montre que Dr. B. a deja recue, et de 750 fr. pour des travaux de maçonnerie faits à la maison de Dr. B. par M. L. depens compensés." When the attendance or report of a medical man is required in any medico-legal case, whether that be of a criminal or civil character, he is entitled to remuneration, according to the following scale:

"Chaque medecin ou chirurgien recevra, savoir—

1mo. Pour chaque visite et rapport, y compris le premier pansement s'il y a lieu—

Dans notre bonne ville de Paris 6 fr.
Dans les villes de quarante mille habitans, et au dessus...... 5 fr.
Dans les autres villes et communes .................. 3 fr.

2do. Pour les ouvertures de cadavres, ou autres operations plus difficiles que la simple visite et en sus des droits ci dessus:

Dans notre bonne ville de Paris 9 fr.
Dans les villes dequarante mille habitans et au dessus ..... 7 fr.
Dans les autres villes et communes .................. 5 fr.

There are some extra allowances, when the medical man is required to perform analytical or other scientific experiments, in cases of suspected poisoning, to report on the state of articles of food, or drink, &c. &c.

Lasty, it is worthy of mention that medical men in France are entitled to demand remunreration, whenever they are cited as witnesses "soit devant le juge d'instruction, soit aux debats, à raison de leurs declarations, visites, ou rapports: les indemnités dues pour cette comparation leur sont alors payées, *comme à des temoins*, et leurs indemnités de route doivent etre les memes que celles des temoins."

We come now to a subject of still more interesting importance to medical men in France, we mean that of certain restrictive or prohibitory laws against their accepting a large donation from a patient during his life, or a large legacy bequeathed in his will. The words of the art. 909 of the civil code are these: "Les docteurs en medecine, ou en chirurgie, les officiers de santé, et les pharmaciens qui auront traité une personne pendant une maladie dont elle meurt, ne pourront profiter des dispositions entre-vifs, ou testimentaires qu' elle aurait faites en leur faveur pendant le cours de cette maladie: sont exceptés, 1, les dispositions remuneratoires faites à titre particulier en egard aux facultés du disposant, et aux services rendus; 2, les dispositions universelles, dans le cas de parenté, jusqu-au quatrieme degré inclusivement, pourvu toutefois que le decedé n'ait pas d'heritiers en ligne directe; à moins que celui au profit de qui la disposition a été faite, ne soit lui meme du nombre de ces heritiers. Les memes regles sont observées à l'egard des ministres des differens cultes." But as in many other cases of legal enactment, the prohibitions contained in the preceding causes may be easily evaded: thus a "donation" may be disguised under "la forme d'une rente," and no objections can be made; or it may be bestowed on the wife, children, or parents of the medical men, and it is equally legitimate; and the same holds true "ou il est etabli que les soins donnés par le medecin l'ont été par suite de l'affection qu'il portait au testateur, et que la liberalité a été determinée aussi par l'affection, que le testateur portait à ce medecin, bien avant sa mort."

So jealous, however, is the law of medical men exerting an undue influence over the minds of patients, that in certain cases of marriage having been contracted during a professional attendance, the husband has been dispossessed of property which may have been left him by his wife. Thus a Dr. Bonnet, in October 1812, married a widow lady, who was then "malade et gisante dans son lit," and indeed suffering from the disease of which she died a month afterwards. By the marriage contract, the survivor was made sole residuary legatee of the other's property. The heirs at law of the wife disputed the legality of the contract; the cause was argued at great length before the "cour royale de Paris," and judgment was at length awarded against the doctor, on the ground that the "donations avaient eté faites dans le cours de la maladie dont il la traitait, et dont elle etait decedée.—*Jurisprudence de la Medecine.*

———

### OF THE DESICCATION OF THE UMBILICAL CORD.

When we are called upon to determine whether an infant has been born alive, or not, it will be found useful to attend to the condition of the umbilical cord among other enquiries. Whenever the cord exhibits the appearances of the ordinary shrivelling, which we observe in a child two or three days after birth —when, for example, it has become of a reddish-brown colour, is flattened and shrivelled, and its vessels are dried and almost obliterated, we may very safely infer that the child has not only been born alive, but has lived for some time after birth. On the other hand, when the cord is pulpy and soft, of a green colour, and presents other traces of putrefaction, the probability is, that the child has been still-born.

The normal desiccation of the cord takes place only during life, and may, therefore, be regarded as a strictly vital process. This sign is well worthy of the attention of the medical jurist, to whose consideration the following conclusions may be acceptable :—1, The desiccation of the umbilical cord takes place only during life—2, from the moment of death, the desiccation is altogether suspended, or proceeds at least very imperfectly—3, whenever the desiccation is considerably advanced, we may conclude that the child had lived for at least one day—4, the cord may indeed be soft, and putrefying, yet the child may have been born alive; but it must have died soon after birth. When the cord putrefies, its outward integument or pellicle generally separates more or less from the inclosed vessels ; but the cord itself does not separate, or become detached from the umbilical ring, as it is known to do when the desiccation has advanced sufficiently far. If, indeed, the body of a still-born infant be kept in circumstances calculated to retard putrefaction, it does sometimes happen that the navel-string becomes dry, and partially shrivelled, and does not exhibit any very obvious appearances of putrefaction ; but the shrivelling is only partial—the cord retains its rounded form, and a certain degree of suppleness.

The following case occurred in the practice of M. Ollivier. He, along with MM. Marc and Denis, was ordered to inspect the body of a full-grown fœtus, which had been dead for at least eight or nine days, and was exposed in the "Morgue." All the organs were in the state of putrefaction—the cavities were distended with gas—the lungs were quite decayed, and the umbilical cord, which seemed never to have been tied, had partaken of the general decomposition ; instead of being dry, flattened, and twisted upon itself, as usually occurs, it had formed at its divided extremity a puckered sac, which resembled a portion of small intestine distended and dried, in consequence of the gelatinous nidus of the vessels having altogether disappeared, and nothing but the investing outer membrane remaining. The epidermis of the abdomen separated easily under the fingers, but the cord was still firmly adherent. The inference from the preceding particulars is, that the umbilical cord, in the present case, did not exhibit the appearances of the normal desiccation, but had undergone a mere putrefaction, like the rest of the body, a process which does not induce a speedy separation of the cord from the umbilical ring. This separation is, in truth, a strictly vital act, and not the effect of simple decay. It is to be observed that the cord is much more tardily affected with putrefaction than the other parts of the body.

The period at which the separation of the cord usually takes place after birth, is from the fourth to the sixth day. Orfila has stated, in his Lectures on Legal Medicine, that the cord begins to desiccate on the first day after birth, and that it usually becomes separated in four or six days. Sometimes there is a slight suppuration round the edges of the umbilical ring, which are then puffy, and somewhat inflamed, before the cord is completely detached; and this is most frequently observed, when the ring or "bourrelet" of integuments advances for a quarter or half an inch on the cord. On the contrary, when this "bourrelet" is small, and not prominent, the separation of the cord is usually dry, and unattended with any traces of suppuration. It is improper, therefore, in medico-legal enquiries, to attach too much importance to the appearance of the umbilical ring, whether it exhibits a redness and other signs of any inflammatory action or not, when we are required to declare if the infant had died before, during, or after delivery. If asked, to what do we attribute the falling off of the cord, we should have no hesitation in replying, that it is the effect of the constriction which the desiccated lymph exerts upon the umbilical vessels, aided by the contractility, or, we should rather say, the tendency to close, of the edges of the ring, a tendency not peculiar to these, but which is invariably observed in all divided living structures.

Haller was certainly most extravagant in referring the separation to a gangrene of the cord; and M. Capuron has, as we have already stated, committed the error of supposing, that it was in all cases the effect of an inflammatory process. In perhaps the majority of cases, there are no traces of any inflammation having ever existed; and, moreover, even if it did always take place, it would not satisfactorily explain the falling off of the cord, as it is an admitted position in pathology, that bloodvessels " au milieu des parties enflammées ne s'enflammaient presque jamais."—*Billard, Traité des Maladies des Enfans.*

---

### Exfoliation of the Epidermis in Infants.

The attention of medical jurists has been directed to this subject also, with the view of determining the probable length of time which a child may have lived after its birth, and M. Orfila has endeavoured to methodize the observations made by preceding authors, as well as those which he has collected in his own experience.

It may be positively asserted that this cuticular exfoliation is so far a vital process, that it occurs only in living children, and, moreover, that it does not begin until after birth. There are no satisfactory examples of the genuine exfoliation having commenced while the fœtus was still in utero. We do not mean to deny the occurrence of separation, or, as it were, unglueing of the epidermis, in cases where a dead child has been retained for some time in the womb; this is well known to be of frequent occurrence, but such separation is essentially distinct, in its features and cause, from the phenomenon which we are at present considering.

M. Orfila has pointed out three different stages of the genuine cuticular exfoliation; the "travail preparatoire," which occurs between the sixth and eleventh days after birth—the "soulevement" of the epidermis, between the end of the third and the middle of the fifth week—and the "exfoliation," which is usually completed between the thirty-fifth and the fortieth days. Such is the result of M. O.'s investigations of this process, as it takes place in healthy children, and when it is not retarded by disease. The late Dr. Billard, who so diligently and so ably pursued the study of infantile diseases, collected a number of observations to test the accuracy of the preceding statements. The result of these was by no means favourable to Orfila's conclusions; for in thirty-two children, in whom the process of exfoliation was " en pleine activité, c'est à dire, de larges ecailles ou de zones très etendues d'epiderme s'enlevaient sur divers points de la surface du corps," 1 was only a day old—7, two days—8, three days—6, four days—6, five days—1, seven days—2, nine days, and 1 fifteen days old.

According to these data, the exfoliation makes the greatest progress between the second and fifth days after birth.

It would be incorrect, however, to lay down any very positive general positions on this subject; for we find that M. Billard states that, in other forty-two infants, whose ages varied from one to ten days, no appearances of the cuticular desquamation were visible; and again, that in some cases, the scales of the epidermis were so very minute, that they fell off in the state of a fine powder, and were indeed not to be observed at all. This is the "exfoliation insensible de l'epiderme" of French authors.

Dr. B. is of opinion that Orfila is far too refinedly minute, when he speaks of three successive stages of the cuticular separation. The "travail preparatoire" is purely fictitious, for no sooner does the epidermis begin to exhibit any clefts or cracks, than a "veritable soulevement" takes place simultaneously; this "soulevement" is either by lines or furrows, by large plates or laminæ, or by furfuraceous scales, in different parts of the surface; thus, the first are generally seen on the skin of the abdomen and of the joints—the second on the chest, back, soles of the feet, &c. and the last on the cheeks, in the armpits, on the shoulders and arms, hips, &c.

With respect to the cause of the cuticular exfoliation in infants, Dr. Billard is inclined to consider it to be the drying which the epidermis, softened and relaxed by having been well soaked for several months in the liquor amnii, undergoes on exposure to the atmosphere. It is to be viewed, therefore, as a mere physical operation of inorganic matter. The new epidermis, it is well known, is extremely tender and delicate, and this circumstance ought always to be attended to in the management and treatment of infants in health, as well as in disease.

It must be unnecessary to caution any medical man against mistaking the separation of the cuticle, the result of putrefaction, from the process to which we have been alluding above. Independently of the other co-existing appearances of decomposition, the offensive smell, the green colour, &c. of the integuments, the peculiar appearance of numerous transparent, colourless, and gelatine-like fibrillæ between the cuticle and cutis vera, which are observed when we cautiously raise up the former membrane, are quite characteristic of putridity.—*Traité des Maladies des Enfans.*

---

MEMORANDA RESPECTING CATARACT.

It has been often remarked, that cataract is one of those diseases, the tendency to which is obviously influenced by hereditary predisposition. The operation of this cause has been distinctly traced in upwards of 30 cases out of 120, admitted by M. Roux within the last few years into the Hôpital de la Charité. The particulars of one case are so curious, that they deserve to be noticed:—A woman, 30 years of age, was affected with cataract; her grandfather, one of her uncles, one of her aunts, and two cousins (all on the father's side), had laboured under this malady, and one of this woman's children was affected with the congenital disease.

M. Roux mentions that, some years ago, he operated for cataract upon three brothers, nearly about the same period; their grandfather, father, and also a younger brother, had all been cataractous.

The important practical question, whether cataract is generally apt to remain confined to one eye for a length of time, or not, is answered in the negative, if we may judge by the results of the 120 patients admitted into the hospital; for, out of this number, there were only two in whom there was but one cataract. We do not mean to assert that the disease was equally advanced in both eyes, but only that, when one eye was fairly cataractous, and had been so for a length of time, there were traces of the disease in the other. M. Maunoir is, therefore, led by these data to believe, that a cataract in one eye (or, perhaps, to speak more correctly, the cause which has produced it) seems to exert, in almost every instance, a hurtful influence upon the other.

The results of the operations (by extraction) have been more favourable to individuals than to single eyes; 73 patients out of 115 recovered their sight—but only 97 operations out of 179 were successful. In thirty of the unsuccessful operations, a secondary cataract, accompanied with or without partial opacity of the cornea, or sometimes with injury and displacement of the pupil, was formed; in fourteen, the eye was destroyed by suppuration; in nineteen the cornea became completely opaque, and in one case the pupil became altogether closed; in the remaining eighteen cases, the lesions were of a complicated kind.

Surgeons have differed in opinion as to the propriety of operating upon both eyes, when cataracous, at the same time; the experience of M. Roux is favourable to the practice, as the proportional success which he obtained in 64 cases in which the double operation was performed at one sitting, was greater than in 48 other cases, in which only one cataract was extracted at a time. The most serious accident which may attend the operation of extraction is, according to the results of the 120 cases, the partial escape of the vitreous humour. In 19 cases in which this unfortunate accident occurred, six only of the patients recovered their sight. The wounding of the iris is a much less unfavourable accident.

It is worthy of notice, that the operation of extraction is that to which M. Roux almost invariably gives the preference; at the Hôtel Dieu, on the contrary, the "abaissement" or couching, is very generally performed.—*Essai sur la Cataracte, par M. Maunoir de Geneve.—Paris,* 1833.

---

### Medical Superstitions in some Parts of France.

A humiliating, but nevertheless an instructive, page of man's absurdities, is furnished by the consideration of the truly ridiculous, and most irrational usages of different countries in the treatment of the prevalent diseases by some of the lower orders. In the mountainous districts of the Haute-Loire, it is a very common custom among the inhabitants to treat pleurisies, pneumonias, and, indeed, almost all active diseases of the chest (and these are by far more frequent than any other class of affections) by applying on the ribs, over the seat of pain, a gutted cock or a tom-cat, if the patient be a male, and a gutted hen or a she-cat if the patient be a female; the correspondence of sex between the medicated and the medicant animal is believed to be quite essential to the perfection of the cure!

The modus operandi of this singular remedy is referred by the Haute-Loirians, to the dead body acting as a magnet to the "mauvaises humeurs de la poitrine," which are supposed to be the cause of the inflammatory disease. After remaining for four and twenty hours applied, it is removed; and, should it then emit an offensive smell, these credulous people imagine that it must necessarily have acted beneficially, and that the incipient decay is solely attributable to the "pourriture" which it has drawn from the patient's body. The above is the first step in the treatment—the second is that of the patient forthwith swallowing the blood of the victim, which has already purified his blood; "tout cela est au moins bien pitoyable;"—truly it is!

Such is one of the methods employed in this rude part of France to remove an alarming disorder. At other times, recourse is had to an "amalgame informe" of melted lard, rancid oil, soot, black pepper, and urine! Well may a Frenchman exclaim—"Ne faut il pas avoir un estomac de cheval pour pouvoir avaler et digerer un ragout aussi horrible:" for our part, we much doubt whether even a horse's stomach could tolerate such a mess. When the enduring powers of the poor patient's stomach are too feeble for this potent medication, a substitute is found in a variety of heating stimulating drinks, prepared with acrid drugs, and with wine, spirits, and pepper. So much for pneumonias, &c.

Phthisis pulmonalis is combated with a savoury julep of goose-dung in white wine; incontinence of urine, with an omelet of roasted rats; peritonitis, with a "boisson" composed of cat's blood and menstrual discharge; tinea capitis, by Gulliver's renowned method of extinguishing fires, viz. "par uriner, quelques jours sur la tete de l'enfant." Severe puerperal after-pains are treated in the Haute-Loire, as indeed in many other countries, by making the women swallow some of the uterine discharges. We need not proceed in our enumeration of such "grossieretes." Satis superque.—*Journal Hebdomadaire.*

---

RHINOPLASTY PERFORMED BY DIEFFENBACH AT THE LA PITIÉ HOSPITAL.

The case was that of a woman, who had lost the greater part of her nose by the destructive effects of a lupous ulceration. M. Dieffenbach happening to be in Paris at the time, was requested by the surgeons of the La Pitie Hospital to perform the operation of rhinoplasty, which, it is well known, he has contributed so much to improve and establish. Having traced on the forehead the shape of the flap which he proposed to employ, (it was of the shape of an ace of clubs, the apex pointing downwards between the superciliary ridges,) he quickly made the necessary incisions, and dissected it from the pericranium; he then divided the integuments covering the root of the nose, pared the edges of the nasal apertures, and "traversé de part en part la racine de la levre" at its union with the septum narium. Having done all this, he proceeded to draw and to keep together as well as was possible, the edges of the wound of the forehead, by means of an interrupted suture.—The pedicle of the flap, reverted or turned once upon itself, was now carefully fitted in between the lips of the incision which had been made through the integuments covering the root of the nose, and several needles were placed above, for the purpose of preventing the pedicle from being in any way displaced. Having completed this, M. D. then fixed the appendix (we suppose that portion of the flap corresponding to the shaft of the spade) intended to form the "sous-cloison" of the new nose, in the opening or incision which had been made through the root of the upper lip, and retained it in this position by means of one or two needles. At this period of the operation, the flap, being held by its two extremities, formed, as it were, a "voile flottant" in front of the nostrils. The remaining steps were the adjusting and retaining in contact, by means of several twisted sutures, the edges of the flap, with the pared edges of the alæ nasi, and the introducing of a small cylinder of rolled lint into each nasal opening, for the purpose of supporting the newly-made member. The exposed ends of the needles, which had been used in making the twisted sutures, were snipped off by strong forceps. The operation lasted for rather more than half an hour. The points to which M. Dieffenbach most strongly directed the attention of the spectators were—1, the necessity of making the appendix, which is to form the "sous-cloison," of a sufficiently ample size, that it may not be destroyed by supervening ulceration or gangrene; 2, the permitting the flap to bleed freely, before it be applied to the nose;—in most cases the oozing will have ceased by the time that the wound of the forehead has been dressed: and 3, the injurious effects of plugging the nostrils with too large rolls of lint after the operation has been completed.—*Journ. Hebdom.*

